# Efficacy and Safety of Resistance Training for Coronary Heart Disease Rehabilitation: A Systematic Review of Randomized Controlled Trials

**DOI:** 10.3389/fcvm.2021.754794

**Published:** 2021-11-05

**Authors:** Yixuan Fan, Meili Yu, Jingen Li, He Zhang, Qiyu Liu, Lin Zhao, Tong Wang, Hao Xu

**Affiliations:** ^1^National Clinical Research Center for Chinese Medicine Cardiology, Xiyuan Hospital, China Academy of Chinese Medical Sciences, Beijing, China; ^2^Graduate School, Beijing University of Chinese Medicine, Beijing, China; ^3^Beijing First Hospital of Integrated Chinese and Western Medicine, Beijing, China; ^4^Dongzhimen Hospital, Beijing University of Chinese Medicine, Beijing, China

**Keywords:** resistance training, coronary heart disease, rehabilitation, systematic review, randomized controlled trials

## Abstract

**Background:** Resistance training (RT), as part of exercise prescriptions during cardiac rehabilitation for patients with cardiovascular disease (CVD), is often used as a supplement to aerobic training (AT). The effectiveness and safety of RT has not been sufficiently confirmed for coronary heart disease (CHD).

**Objective:** To provide updated evidence from randomized clinical trials (RCTs) on efficacy and safety of RT for the rehabilitation of CHD.

**Method:** Three English and four Chinese electronic literature databases were searched comprehensively from establishment of each individual database to *Dec, 2020*. RCTs which compared RT with AT, no treatment, health education, physical therapy, conventional medical treatment (or called usually care, UC) in CHD were included. Methodological quality of RCTs extracted according to the risk of bias tool described in the Cochrane handbook. The primary outcomes were the index of cardiopulmonary exercise testing and the quality of life (QOL). The secondary outcomes included the skeletal muscle strength, aerobic capacity, left ventricular function and structure.

**Results:** Thirty-right RCTs with a total of 2,465 participants were included in the review. The pooling results suggest the RT+AT is more effective in the cardiopulmonary exercise function (peak oxygen uptake, peak VO_2_) [MD, 1.36; 95% CI, 0.40–2.31, *P* = 0.005; *I*^2^ = *81%, P* < 0.00001], the physical score of QOL [SMD, 0.71; 95% CI, 0.33–1.08, *P* = 0.0003; *I*^2^ = 74%, *P* < 0.0001] and global score of QOL [SMD, 0.78; 95% CI, 0.43–1.14, *P* < 0.0001; *I*^2^ = 60%, *P* = 0.03], also in the skeletal muscle strength, the aerobic capacity and the left ventricular ejection fraction (LVEF) than AT group. However, there is insufficient evidence confirmed that RT+AT can improve the emotional score of QOL [SMD, 0.27; 95% CI, −0.08 to 0.61, *P* = 0.13; *I*^2^ = 70%, *P* = 0.0004] and decrease left ventricular end-diastolic dimension (LVEDD). No significant difference between RT and AT on increasing peak VO_2_ [MD, 2.07; 95% CI, −1.96 to 6.09, *P* = 0.31; *I*^2^ = 97%, *P* < 0.00001], the physical [SMD, 0.18; 95% CI, −0.08 to 0.43, *P* = 0.18; *I*^2^ = 0%, *P* = 0.51] and emotional [SMD, 0.22; 95% CI, −0.15 to 0.59, *P* = 0.24; *I*^2^ = 26%, *P* = 0.25] score of QOL. Moreover, the pooled data of results suggest that RT is more beneficial in increasing peak VO_2_ [MD, 3.10; 95% CI, 2.52–3.68, *P* < 0.00001], physical component [SMD, 0.85; 95% CI, 0.57–1.14, *P* < 0.00001; *I*^2^ = 0%, *P* = 0.64] and the emotional conditions [SMD, 0.74; 95% CI, 0.31–1.18, *P* = 0.0009; *I*^2^ = 58%, *P* = 0.12] of QOL and LVEF, and decreasing LVEDD than UC. Low quality evidence provided that RT had effect in decreasing rehospitalization events than UC [RR, 0.33, 95% CI 0.17 to 0.62, *P* = 0.0006; *I*^2^ = 0%, *P* = 0.64]. There is no significant difference in the safety of RT compared to AT.

**Conclusions:** RT combined with AT is more beneficial than AT alone for CHD. RT can effectively improve the capacity of exercise and the QOL compared with UC. But the difference between RT and AT is still unknown. More high-quality and large-sample studies are needed to confirm our findings.

## Introduction

Coronary heart disease (CHD), a highly prevalent chronic disease, is the major cause of death and disability worldwide (1). According to statistics, the number of patients with CHD is raising in various countries nowadays. In US, approximately 20.1 million adults nationwide had CHD, which had become the most common cause of death from cardiovascular disease (CVD) (2). About $8.7 billion was spent on hospitalizations for CHD in America in 2017 (3). CHD places a huge economic and social burden on the society.

The key to the treatment of CHD is to reduce cardiovascular risk factors and to improve long-term prognosis. Physical inactivity is a universally recognized risk factor, and the burden of physical inactivity related death caused by CHD is estimated to be 9.9% (4). Current studies have found that physical activity can effectively increase coronary blood flow and reduce cardiovascular mortality in patients with CHD (5–7). Exercise, similar to compounded medications, can be beneficial to the recovery of patients with CHD in multiple areas (8). Exercise-based cardiac rehabilitation has been included as an important element of cardiac rehabilitation (CR) in the primary and secondary prevention of CHD (9–11). Three types of exercise are included in CR, namely aerobic training (AT), resistance training (RT, also called strength training) and flexibility training. RT or strength training has multiple positive effects on cardiovascular health. Weighted dumbbells, elastic bands, lifting machines, or people training with own body weight for resistance. At present, studies have found that RT reduces cardiovascular mortality, prevents obesity, reduces blood pressure, and improves insulin resistance (8, 9, 12–16). On the other hand, RT is not clearly recommended in primary prevention of cardiovascular disease due to insufficient evidence of RT reducing risk of the arteriosclerotic cardiovascular disease (9, 17). In addition, there is insufficient evidence to confirm that RT is as safe as AT (9, 18).

Since peak oxygen uptake (peak VO_2_) is strongly associated with cardiovascular risk, it is often used as a primary effect indicator to evaluate RT interventions (19). Previous meta-analysis showed that RT or RT plus AT can increase peak VO_2_ and muscle strength in patients with CHD effectively, but these reviews have not been updated (20, 21) and the effect of RT alone was not reflected. Meanwhile, evidence of RT on quality of life and mental health, also important risk and prognostic factor for CHD (22, 23), is insufficient. A meta-analysis found that RT had a beneficial effect on quality of life, but this analysis just including 9 articles, which lack of adequate evidence (24). RT has not been played the main role in exercise prescriptions for patients of CVD (25). Therefore, we conducted a meta-analysis with a comprehensive search to evaluate the effects of RT on exercise capacity, quality of life (QOL), cardiac function and safety of CHD. The purpose of our study is to explore the role of RT in the rehabilitation prescription of patients with CHD.

## Methods

### Study Registration

This study was registered in the international prospective register of systematic reviews (PROSPERO registration number CRD42021233033) and was conducted following the Preferred Reporting Items for Systematic Reviews and Meta-Analyses (PRISMA) statement. Please find the detail of the protocol at https://www.crd.york.ac.uk/PROSPERO/#myprosperoID=CRD42021233033.

### Criteria for Inclusion and Exclusion

In our analysis, the criteria for inclusion were the following: (a) the studies were randomized clinical trials (RCTs); (b) no restrictions were put on the language of the literature; (c) the patients and control subjects should have CHD (including myocardial infarction, coronary artery bypass grafting, percutaneous transluminal coronary angioplasty, percutaneous transluminal coronary intervention, angina or other CHD types) with any population characteristics; (d) RT with other treatments, such as AT, no treatment, health education, physical therapy, conventional medical treatment, compared to the same treatments were included; (e) the studies have found control groups for comparing different interventions; and (f) the index of the cardiopulmonary exercise testing, such as the maximum rate of oxygen uptake (VO_2_ max) or peak VO_2_, and the quality of life (QOL), assessed by relevant standard scales, was also included as one of primary outcomes. The secondary outcomes included the patients' skeletal muscle strength, aerobic capacity with anaerobic threshold as the main indicator; left ventricular function and structure assessed by resting echocardiography, mainly left ventricular ejection fraction (LVEF) and left ventricular end-diastolic dimension (LVEDD). Adverse events were reported as the safety outcomes. At least one of the primary outcomes or secondary outcomes or adverse events should be reported in the included trials.

Studies would be excluded if (a) it would not be included if the article was not full text on electronic databases for published studies; (b) subjects were disability or suffered from other diseases to research; (c) subjects were disability or suffered from other diseases to research; (d) interventions were not evaluated the post-intervention effects of RT.

### Search Strategy

We conducted an up-to-date comprehensive search in four Chinese databases and three English databases, including PubMed, Cochrane Library, Embase, Chinese National Knowledge Infrastructure Databases (CNKI), Chinese Biomedical Literature Database (SinoMed), Chongqing VIP Chinese Science and Technology Periodical Database (VIP) and Wanfang Database. Search time from establishment of each individual database to *Dec, 2020*. The search terms of retrieval strategy are slightly different based on each database, including “resistance,” “resistance training,” “strength exercise,” and “randomized controlled trials.” In English databases, we added the main MeSH terms as keywords for part of search strategy. The detailed search strategy and results is presented in Appendix 1.

### Study Selection

Two authors (Yixuan Fan and Meili Yu) independently screened the retrieved records by the same selection criteria in the Note Express 3.2. Non-relevant and repeated studies were removed by reviewing the titles and abstracts. All articles or RCTs' data with potentially relevant trials were downloaded and reviewed before final inclusion. Disagreements were resolved by consultation with a third investigator (Hao Xu).

### Data Extraction and Management

Two reviewers (Yixuan Fan and Jingen Li) independently extracted the data from included studies. A standardized data extraction form was used to extract data, including the author names, year of publication, regions, diseases, age and sex of the participants, interventions, disease duration, outcomes, course of interventions, and follow-up. The data were imported into an electronic data sheet by the two reviewers individually. Disagreements were resolved by consensus, including a third investigator (Hao Xu). If the data in these RCTs were missing or not recorded completely, we attempted to contact the authors for further information.

### Quality Assessment

Two authors (Meili Yu and He Zhang) independently assessed the methodological quality of included trials. Methodological quality of RCTs extracted according to the risk of bias tool described in the Cochrane handbook for systematic reviews of interventions (26). Seven elements were assessed: random sequence generation, allocation concealment, blinding of participants and personnel, blinding of outcome assessment, incomplete outcome data, selective reporting, and other bias (whether funds or institutions supported the study). The third author evaluated the results that remained disputable.

### Data Analysis

All statistical analysis were performed using RevMan 5.3 (The Cochrane Collaboration) software. Date was summarized using risk ratio (RR) with its 95% confidence interval (CI) for binary outcomes and mean difference (MD) or standard mean difference (SMD) with 95% (CI) for continuous outcomes. SMD eliminates the influence of the absolute value of a study and eliminates the influence of the measurement unit on the results. Statistical heterogeneity among included trials were evaluated by *I*^2^-test. If *I*^2^-value is <50%, we used fixed effect model to pool the data. If *I*^2^-value was equal to or more than 50% (*I*^2^ ≥ *50%*), random effects model was used in meta-analysis (27, 28). Funnel plots were used to explore the possibility of small study effects or publication bias, if there are 10 or more studies in an analysis. Publication bias was tested visually using Egger's regression asymmetry test (29). Subgroup analyses were conducted to determine the evidence according to different control measures (e.g., only resistance training, combine resistance and aerobic training, resistance training and flexibility and balance exercises), different types of resistance training (e.g., push-ups, hand weights, elastic bands, weight machines), and different lengths of rehabilitation intervention, etc.

Sensitive analyses were conducted for all significant findings by using Stata 12.0 software to assess the robustness of the primary analysis. And these significant outcomes also were tested by using Egger's regression asymmetry test to judge whether there is high risk of bias.

## Results

### Results of the Search

**Figure 1:**
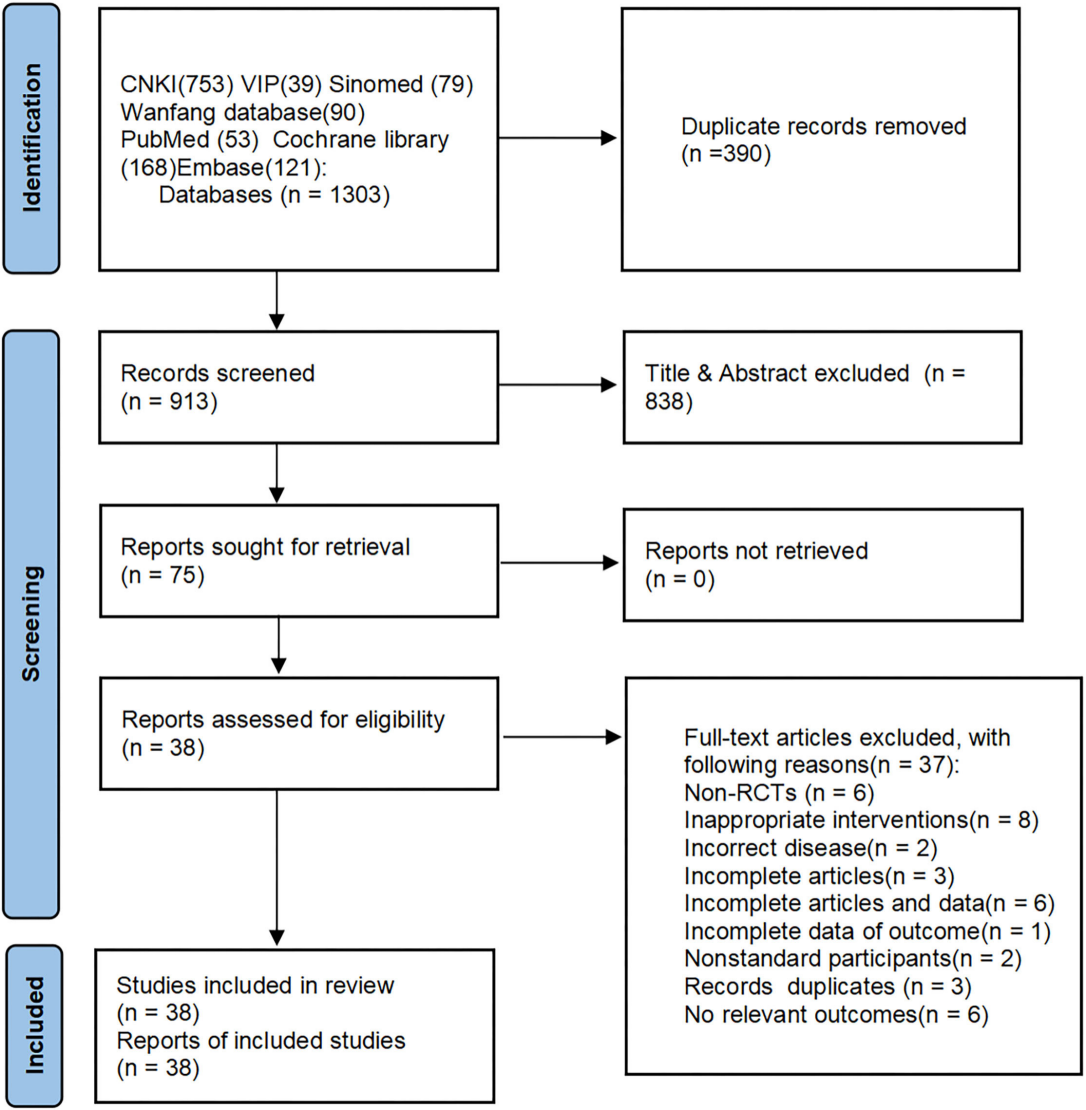
The flow chart of study searching and screening.

After a primary search of 3 English databases and 4 Chinese databases, we got 1,303 citations for further evaluation. Full-text articles of 75 trials were assessed, and 38 RCTs were eligible and included in this review The flow chart of study searching and screening is shown in Figure 1.

### Description of the Included Studies

Thirty-seven of the included trials were published in Chinese and English (30–66) and 4 of these had been registered on the website (33–35, 67) with a total of 2,465 participants (1,178 in the intervention group and 1,287 in the control group), including 1,468 men and 893 women (2 articles no mention) (55, 59). The sample sizes of all included trials varied from 16 to 200 participants (8 to 100 participants in each group). Diseases included post coronary artery bypass grafting (CABG) [or called coronary artery bypass surgery (CABS)], myocardial infarction (MI), percutaneous transluminal coronary intervention (PCI), acute myocardial infarction (AMI), myocardial revascularization surgery (MRS), stable coronary artery disease (SCAD), percutaneous transluminal coronary angioplasty (PTCA) and so on. There was a wide variation in the age range (27–86 years) and disease duration (11 days to 25 years) of included trials.

RT or strength training were used alone or combined with AT in the included trials. Thirty (30–58, 67) of the 38 trials compared RT plus AT with AT alone (RT+AT vs. AT). Three trials (43, 59, 60) compared RT vs. AT. And seven trials (43, 61–66) were RT vs. usual care (UC) including daily routines (61, 62, 64–66), drug treatments (62–64) or no training (43). The treatment duration ranged from 3 weeks to 1 year. Four trials (33–35, 67) were registered and were provided with registration numbers. RT modes included extremities exercises against persons' own weight (such as biceps curl, leg extension and so on) (27 trials) (30, 31, 33–47, 49–52, 56–59, 61, 67), elastic bands (12 trials) (32, 43, 48, 50, 51, 53, 54, 60, 62–66), dumbbells (50, 51, 55), and lower extremity sports rehabilitation apparatus (64). The intensity of RT ranged from 15 to 85% of 1 repetition maximum (1RM), and most of the tests have a gradual increase in the intensity of motion. Sixteen trials (32–34, 37, 39, 41–43, 46, 47, 50, 52–54, 59, 60) reported peak VO_2_ as the outcome measure, and two trials reported VO_2_ max (48, 57) as the result of the cardiopulmonary exercise testing. The VO_2_ max also represents the biggest shoot of the amount of oxygen that can be achieved as much as possible under a certain type of exercise. Therefore, the actual peak VO_2_ is similar to the VO_2_ max (68). Eleven trials (30, 32, 34, 45, 46, 59, 61, 62, 64, 65, 67) reported the data of adverse events and the remaining 14 trials (31, 37, 39, 46–54, 60, 66) mentioned no adverse events. Thirteen trials (31, 33, 36–40, 42, 44, 47–49, 63) did not mention adverse reactions. Eight trials (34, 36, 37, 39, 43, 44, 64, 67) mentioned the follow-up duration of posttreatment, which varied from 6 weeks to 1 years. The characteristics of other details of all trials are summarized in Appendix 2.

### Risk of Bias of Included Trials

**Figure 2:**
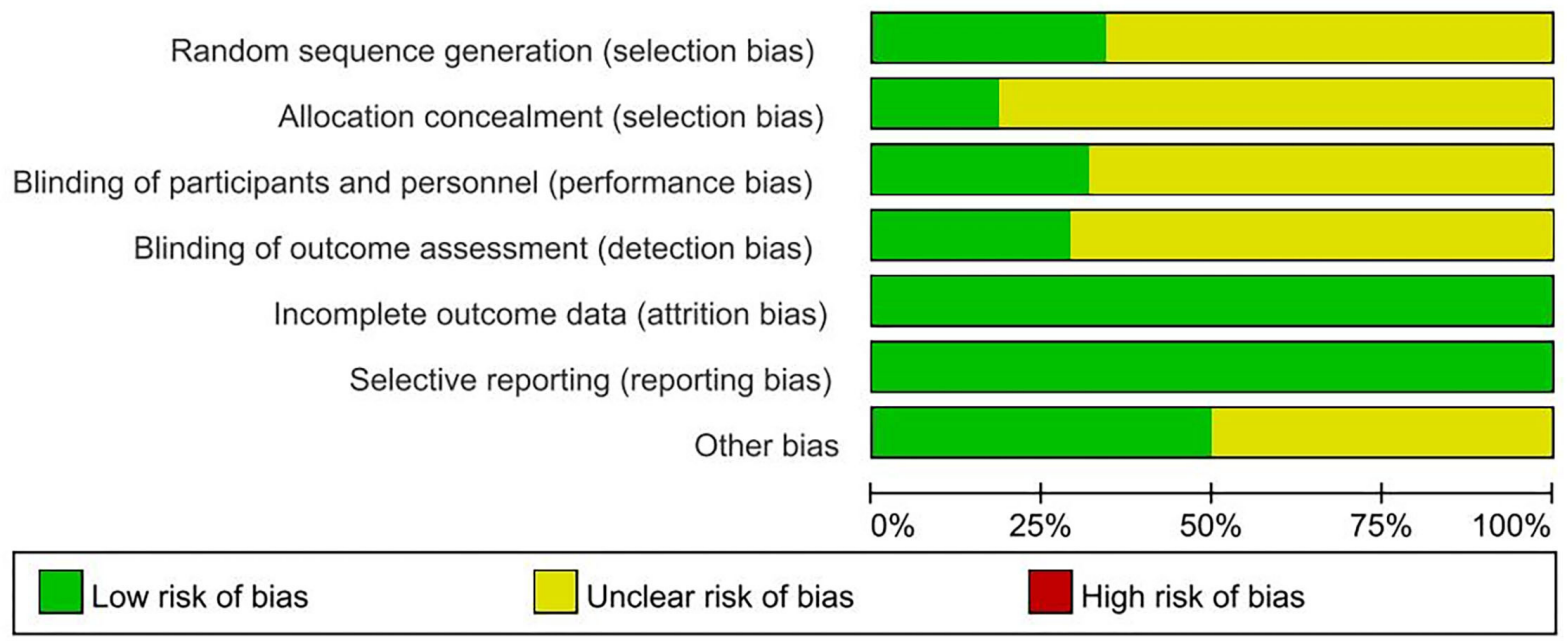
Risk of bias graph of 73 included trials.

Nine (33, 37, 48, 50, 51, 57, 58, 64, 66) of the included trials were judged as high quality (at least four of seven items were assessed as low risk of bias, in which strictly designed with randomization must be included). Thirteen (32, 33, 36, 37, 48–51, 57, 58, 64–66) of included trials used proper methods to generate randomization sequence. Seven trials (34, 35, 37, 50, 51, 54, 66) described methods of allocation concealment. Twelve trials (30, 34, 35, 37, 43, 47, 50, 51, 57, 60, 64, 67) reported blinding methods clearly in the articles or the protocol were assessed as low risk of bias on blinding to participants and personnel. Eleven trials (30, 33, 35, 37, 43, 50, 51, 58, 60, 64) used the third party for analysis to evaluate the outcome indicators were assessed as low risk of bias on blinding of outcome assessors. All trials (30–67) were assessed as low risk of bias on the item of incomplete outcome data. Though there were dropouts excluded from analyses, authors have explained the reasons for the patients' withdrawal from the test and whether these patients' data were included in the analysis results. Protocol of most included trials could not be accessible, but we assessed them as having low risk of reporting bias, since they all reported the important outcome measurements after RT interventions. Nineteen trials (30, 33–35, 37, 40, 43, 46–48, 50, 51, 55, 57–59, 62, 64, 67) were assessed as low risk of other bias by describing the funding issue or no conflict of interest clearly, while the remaining trials not reporting the above information were assessed as an unclear risk of other bias. Risk of bias graph of 38 included trials is shown in Figure 2.

### Estimate the Efficacy and Safety of RT for the Rehabilitation in CHD

#### Comparison 1: RT+AT vs. AT

##### Primary Outcome

###### Peak VO_2_ and VO_2_ max.

**Figure 3:**
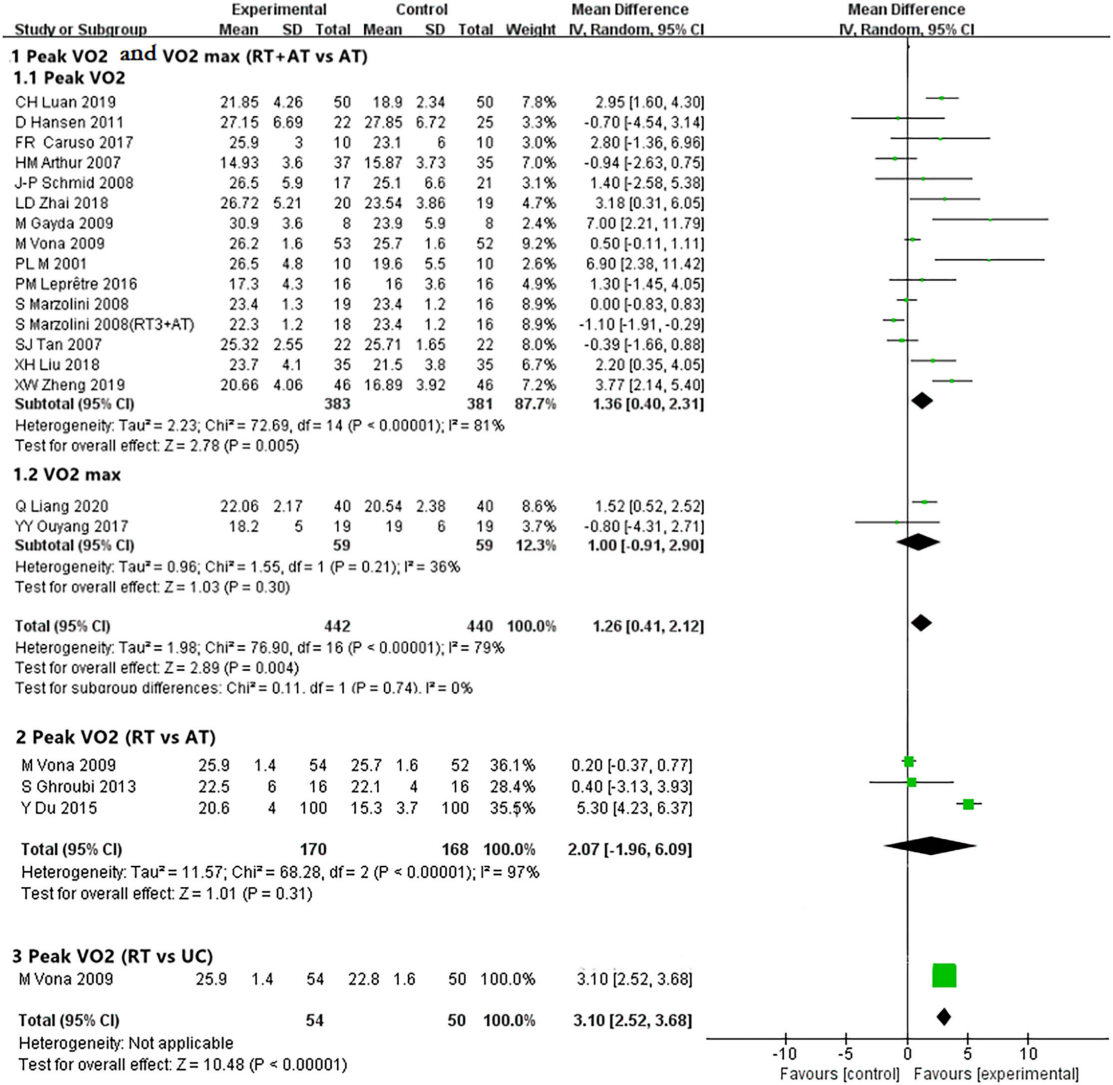
Forest plot showing peak VO2 and VO2 max.

Peak VO_2_ (33–35, 39, 41– 43, 46, 47, 50, 52–54) and VO_2_ max (50, 57) were selected as the main outcome indicators for the index of the cardiopulmonary exercise testing. One study (50) was analyzed twice because of the varied sets of RT (3 sets of resistance training or 1 set of resistance training). Pooled results showed 14 trials (32–35, 39, 41–43, 46, 47, 50, 52–54) involving 764 participants showed RT compared with AT had significantly better effect than AT alone in improving VO_2_ peak [MD, 1.36; 95% CI, 0.40–2.31, *P* = 0.005; *I*^2^ = 81%, *P* < 0.00001 (the *P*-value obtained from the Chi-square test, the same below)]. However, pooled results from 2 trials (48, 57) involving 118 participants showed no difference between RT plus AT and AT alone on VO_2_ max (ml/kg/min) [MD, 1.00; 95% CI, −0.91 to 2.90, *P* = 0.30; *I*^2^ = 36%, *P* = 0.21] (Figure 3, details data presented in Appendix 4).

###### Quality of Life.

**Figure 4:**
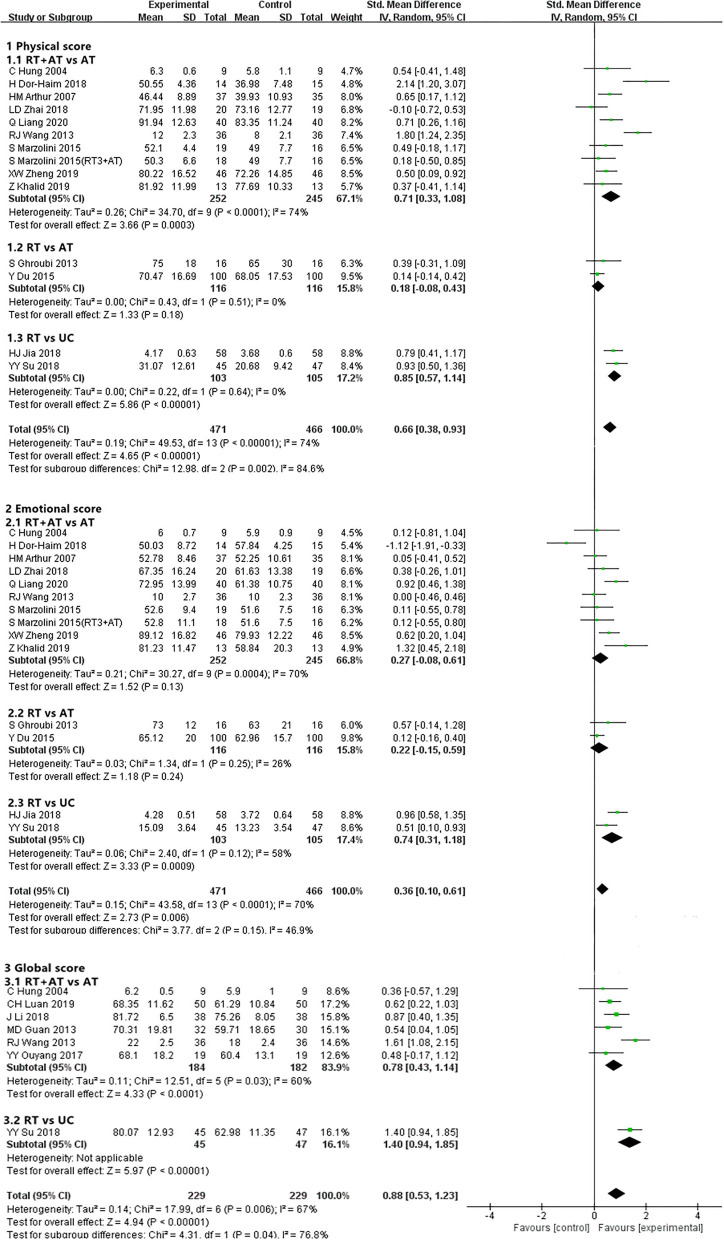
Forest plot showing QOL of RT for CHD.

Nine (31, 35, 37, 41, 48, 49, 51, 54, 58) of 38 trials involving 481 participants reported the physical and emotional score. Compared AT alone, RT plus AT, had better effect in increasing the physical score of QOL [SMD, 0.71; 95% CI, 0.33–1.08, *P* = 0.0003; *I*^2^ = 74%, *P* < 0.0001]. However, no significant difference between the 2 interventions in emotional score [SMD, 0.27; 95% CI, −0.08 to 0.61, *P* = 0.13; *I*^2^ = 70%, *P* = 0.0004]. One study (51) was analyzed twice because of the varied frequency of RT (3 times and 1 time of RT). Six trials assessing global score (31, 32, 38, 44, 49, 57) including 366 patients of CHD compared RT plus AT vs. AT alone. The pooled results of meta-analysis showed RT+AT had a significantly greater effect than AT [SMD, 0.78; 95% CI, 0.43–1.14, *P* < 0.0001; *I*^2^ = 60%, P = 0.03] (Figure 4 and Appendix 4).

##### Secondary Outcomes

###### Skeletal Muscle Strength.

Even though most of included trials had measured many items to evaluate interventions of the skeletal muscle strength, we chose 6 projects of all which can indicate the strength of the upper and lower limbs and torso as the outcomes. And these trials were only the comparison of RT combined with AT vs. AT alone.

According to our subgroup analysis of results, the pooled results showed 4 trials (31, 40, 45, 46) involving 110 participants showed RT+AT had some improvement than AT alone in shoulder press (kg) [SMD, 0.79; 95% CI, 0.06–1.52, *P* = 0.03; *I*^2^ = 68%, *P* = 0.02]. Pooling results of 3 trials (31, 40, 46) with 70 patients of CHD showed RT+AT had a slightly better effect on increasing biceps curl (kg) than AT [SMD, 0.97; 95% CI, 0.47–1.48, *P* = 0.0001; *I*^2^ = 0%, *P* = 0.53]. The fixed effects model outcome of meta-analysis reported that 4 trials (30, 31, 40, 46) including 104 patients indicated there was a greater meaningful effect in increasing chest press (kg) in the RT+AT group [SMD, 0.96; 95% CI, 0.55–0.37, *P* < 0.00001; *I*^2^ = 0%, *P* = 0.59]. Meta-analysis of 5 trials (37, 45, 49, 52, 53) with 298 patients showed compared with AT groups, RT+AT improved arm flexion (kg) [SMD, 0.45; 95% CI, 0.22–0.68, *P* = 0.0002; *I*^2^ = 0%, *P* = 0.87] as well as leg flexion (kg) [SMD, 0.96; 95% CI, 0.38–1.55, *P* = 0.001; *I*^2^ = 83%, *P* < 0.0001] more significantly. We performed meta-analysis for 6 trials (30, 45, 46, 49, 52, 53) with 280 participants, which suggested that RT+AT was slightly superior to AT in improving knee extension (kg) than AT [SMD, 0.90; 95% CI, 0.65–1.16, *P* < 0.00001; *I*^2^ = 5%, *P* = 0.39] (Appendix 4).

###### Aerobic Capacity.

The anaerobic threshold, as the threshold from AT to anaerobic exercise, can be used as one of the main indicators to evaluate aerobic capacity (68, 69). Based on meta-analysis in interventions of RT+AT vs. AT including 4 trials (32, 48, 53, 54) with 342 subjects reported RT+AT were more effective in raising the anaerobic threshold (ml/kg/min) [MD, 1.61; 95% CI, 0.91–2.31, *P* < 0.00001; *I*^2^ = 54%, *P* = 0.09] (Appendix 4).

###### Left Ventricular Function and Structure.

Meta-analysis of 9 trials (32, 36, 39, 48, 52, 54–57) involving 518 participants showed RT+AT had more significant improvement in LVEF (%) than AT alone [MD, 4.17; 95% CI, 2.15–6.19, *P* < 0.0001; *I*^2^ = 49%, *P* = 0.05]. But there was no difference in effect between RT plus AT and AT in decreasing LVEDD (mm), which including 3 trials (45, 54, 55) with 194 participants [MD, −4.53; 95% CI, −11.55 to 2.48, *P* = 0.21; *I*^2^ = 93%, *P* < 0.00001] (Appendix 4).

#### Comparison 2: RT vs. AT

##### Primary Outcome

###### Peak VO_2_.

Three trials (43, 59, 60) involving 338 patients of CHD compared RT with AT and no significant difference between 2 groups [MD, 2.07; 95% CI, −1.96 to 6.09, *P* = 0.31; *I*^2^ = 97%, *P* < 0.00001] (Figure 3 and Appendix 4).

###### Quality of Life.

Two trials (59, 60) involving 232 participants reported physical and emotional score of QOL. Pooled results showed that RT is similar with AT on physical component [SMD, 0.18; 95% CI, −0.08 to 0.43, *P* = 0.18; *I*^2^ = 0%, *P* = 0.51] and the emotional component of QOL score [SMD, 0.22; 95% CI, −0.15 to 0.59, *P* = 0.24; *I*^2^ = 26%, *P* = 0.25] (Figure 4 and Appendix 4).

##### Secondary Outcomes

###### Anaerobic Threshold.

Only one trials (60) with 200 patients measured anaerobic threshold (ml/kg/min) and reported RT alone had a greater beneficial effect than AT [MD, 2.40; 95% CI, 1.50–3.30, *P* < 0.00001] (Appendix 4).

###### LVEF.

The benefit of RT alone for LVEF (%) was more significantly improved than AT in the analysis of 1 trial (60) involving 200 participants [MD, 4.60; 95% CI, 2.52–6.68, *P* < 0.0001] (Appendix 4).

#### Comparison 3: RT vs. UC

##### Primary Outcome

###### Peak VO_2_.

Even through only 1 trial (43) involving 104 patients compared the RT group with the no training group, the meta-analysis found a clinically meaningful effect on RT in improving the peak VO_2_ (ml/kg/min) [MD, 3.10; 95% CI, 2.52–3.68, *P* < 0.00001] (Figure 3 and Appendix 4).

###### Quality of Life.

Pooled results from 2 trials (61, 66) involving 208 participants showed that RT had a greater improvement in the physical conditions [SMD, 0.85; 95% CI, 0.57–1.14, *P* < 0.00001; *I*^2^ = 0%, *P* = 0.64] and the emotional conditions [SMD, 0.74; 95% CI, 0.31–1.18, *P* = 0.0009; *I*^2^ = 58%, *P* = 0.12] of QOL than daily routines. Similarly, higher score in the global part of QOL under RT approached in the 1 trial (66) involving 92 participants compared with daily routines [SMD, 1.40; 95% CI, 0.94–1.85, *P* < 0.00001] (Figure 4 and Appendix 4).

##### Secondary Outcomes. Left Ventricular Function and Structure

Three trials (61, 63, 65) involving 236 participants investigated the changes of LVEF (%) between RT and daily routines or drug treatments. Pooling result showed the significantly better recovery in LVEF after RT [MD, 7.65; 95% CI, 3.73–11.57, *P* = 0.0001; *I*^2^ = 82%, *P* = 0.004] (Appendix 4). There was a more significant decrease of LVEDD (mm) in RT than daily routines by pooling results of 2 trials (61, 65) involving 196 participants [MD, −6.61; 95% CI, −11.33 to −1.88, *P* = 0.006; *I*^2^ = 93%, *P* = 0.0002] (Appendix 4).

### Adverse Events

Eleven trials (30, 32, 34, 45, 46, 59, 61, 62, 64, 65, 67) reported adverse events. Of these, 10 trials (30, 32, 34, 45, 46, 59, 61, 62, 64, 67) reported some adverse events (such as knee pain, significant exercise-induced ST-segment depression without chest pain rather than readmission) and 3 trials (29, 60, 61) reported rehospitalization.

Pooled result of 6 trials (30, 32, 34, 45, 46, 67) involving 333 participants reported RT+AT and AT had no significant difference in some adverse events [RR 1.39, 95% CI 0.28–6.78, *P* = 0.69; *I*^2^ = 49%, *P* = 0.08]. Only one trial (59) involving 32 participants compared RT with AT in some adverse events, and the pooling result showed there were no difference between 2 interventions [RR 0.43, 95% CI 0.13–1.37, *P* = 0.15]. Pooling result of 3 trials (61, 62, 64) of 10 including 247 participants reported RT had no difference compared with drug treatment and daily routines [RR 0.49, 95% CI 0.23–1.07, *P* = 0.07; *I*^2^ = 89%, *P* < 0.0001] (Appendix 5).

One trial (32) of 3 including 100 participants compared RT+AT with AT in rehospitalization and there was no difference in the pooling result [RR 0.50, 95% CI 0.05–5.34, *P* = 0.57]. The available pooled result of the remaining 2 trials (64, 65) including 147 patients of CHD showed RT had beneficial effect in decreasing rehospitalization events than daily routines and drug treatment [RR 0.33, 95% CI 0.17–0.62, *P* = 0.0006; *I*^2^ = 0%, *P* = 0.64] (Appendix 5). The details of total adverse events data were listed in Appendix 5.

### Sensitivity Analysis

The sensitivity analyses were performed by excluding trials included in each outcome on a case-by-case basis. It was stable for the significance of all ending indicators including the primary outcome, the secondary outcomes and adverse events.

### Heterogeneity and Subgroup Analysis of Outcomes

The heterogeneity of some results changed markedly when we eliminated the tests one by one. Although meta-regression analysis was performed to explore the sources of this high between-study heterogeneity, we could not find the detailed sources of heterogeneity due to meta-regression analysis concerning the relationship between RT+AT with AT group and the peak VO_2_. Subgroups were explored for different nationalities, ages, and sub-clinical diagnosis, which did not reduce heterogeneity. To investigate the source of heterogeneity for the physical of QOL in the RT+AT with AT group, we did subgroup analyses and found that the heterogeneity originated from different evaluation scales. A subgroup analysis of trials (31, 37, 41, 48, 51, 54, 58) measured using the SF-36 scale and the MacNew heart disease health-related QOL instrument revealed 0 in heterogeneity, which was also in analysis for the remaining trials.

The heterogeneity of peak VO_2_ (or VO_2_ max) in the subgroup of RT vs. AT was changed from over 50% to zero, which happened with the trial (60) excluded. In the shoulder press subgroup, heterogeneity clearly disappeared with the elimination of this trial (45). The trial (49) in the RT+AT vs. AT significantly influenced the heterogeneity of the global score of QOL. When we excluded the trial (54) in the RT+AT vs. AT subgroup, the heterogeneity of anaerobic threshold had changed significantly, also in the same subgroup of LVEDD. And the elimination of this trial (65) could beneficial decreasing the heterogeneity of LVEF. The heterogeneity during the some adverse events had been changed effectively after the trial (62) excluded. However, exclusion of these trials didn't affect the stability of the results. The further subgroup analysis could not be performed because there were not sufficient numbers of trials in the individual outcome groups already analyzed.

### Assessment for Publication Bias

**Figure 5:**
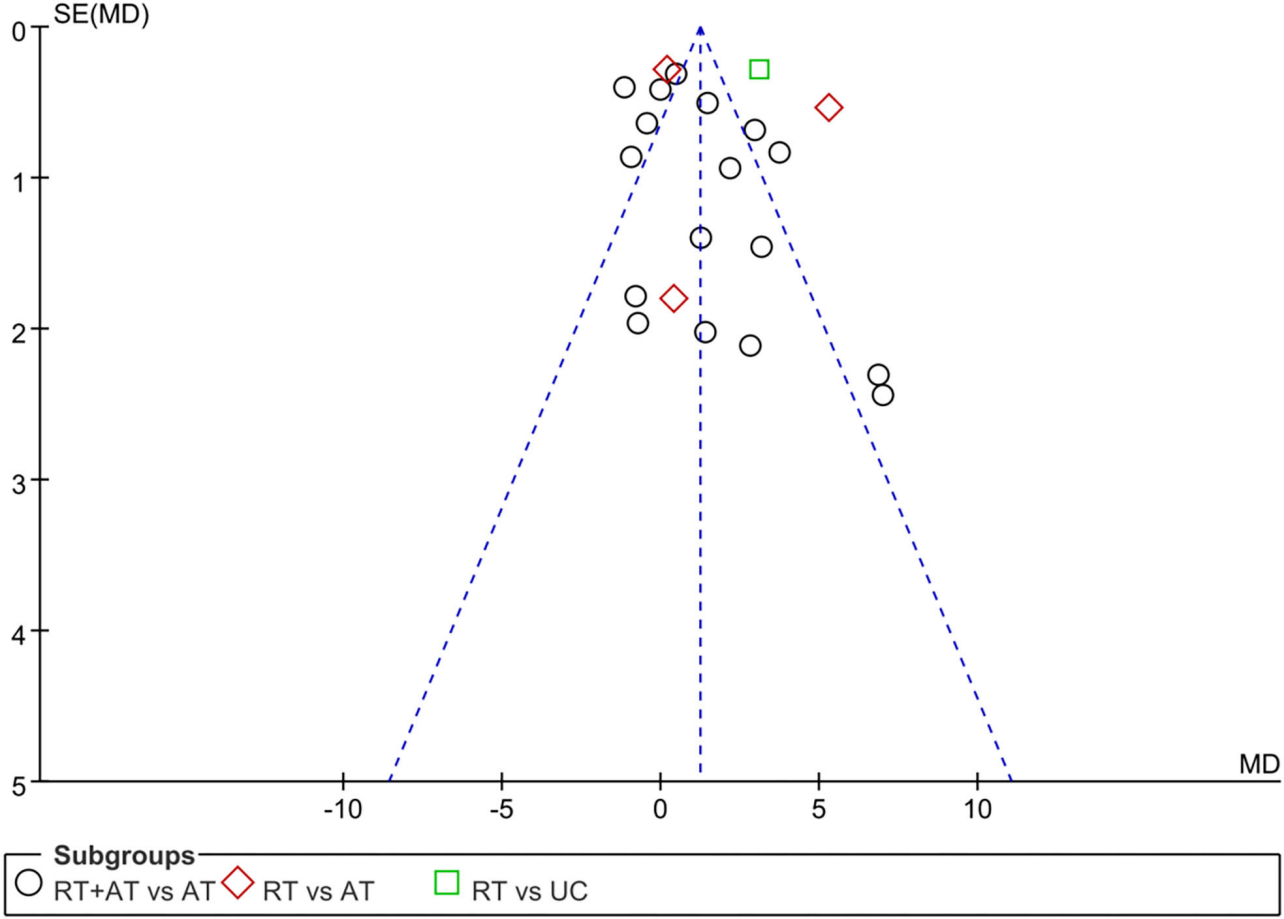
Funnel plot of publication bias.

The funnel plot of summarized 18 RCTs (33, 34, 39, 41, 42, 45–48, 50, 52–54, 57, 59, 60) which reported the primary outcome. It was generally symmetrical representing a low risk of publication bias as shown in Figure 5. The Egger's test outcome showed there was no publication bias (*P* = 0.477 > 0.05).

## Discussion

In this study, we analyzed the association between RT and CHD rehabilitation using a meta-analysis to obtain a powerful conclusion. A total of 38 RCTs (30–67) involving 2,465 patients with a diagnosis of CHD were included by searching on electronic literature databases.

Our meta-analysis results of 3 comparisons suggest the RT+AT is more effective in the cardiopulmonary exercise function, the physical and global component of QOL, the skeletal muscle strength, the aerobic capacity and left ventricular function (the LVEF indicator) than AT group. Compared with AT, there is insufficient evidence that RT+AT can improve the emotional of QOL score and decrease LVEDD. No significant difference between RT and AT on increasing peak VO_2_, the physical and emotional score of QOL. Only 1 trial reported that RT was superior to AT in improvement of anaerobic threshold and LVEF. Definitely, the pooled data of results suggest that RT is more beneficial in increasing peak VO_2_, physical and emotional component of QOL and LVEF, and decreasing LVEDD than UC (daily routines, drug treatments, or no training). Only one trial showed a significant improvement in global QOL scores with RT compared to UC. One trial (30) reported the result of flexibility for CHD's patients, while there was no difference between the RT alone group and the flexibility training group.

According to our statistics, pooling results showed no significant difference in adverse events among the 3 comparisons except reducing rehospitalization for RT compared to UC.

In our review, the heterogeneity of most outcomes were reducing after sensitivity or subgroup analysis, expect for the peak VO_2_ in the RT+AT with AT subgroup. And our literature for the primary outcome was no publication bias by analysis and the Egger's test.

### Strength and Limitation

To our knowledge, our analysis of combined RT+AT with AT is consistent with the results of a previous meta-analysis in increasing peak VO_2_ or VO_2_ max (21, 70, 71), physical score of QOL (72), muscle strength (20, 21, 70–72), anaerobic threshold (70, 72). It is a timely update of the previous study (23) to assess effect of RT on CHD and the pooling results of our meta-analysis provided moderate evidence. Meanwhile, it is the first time to analyze the QOL of participants after training for RT, which concentrated on not only the physical fitness but also the mental health for the patients with CHD. After all, negative emotions are also one of the reasons for the aggravation of CHD (24). Even though the included trial concentrated on comparing RT+AT and AT, our study still had 3 subgroups to investigate the effect of intervention of RT alone.

Our study has a few limitations of the meta-analysis. First, though we have tried our best to conduct a comprehensive search, the quality of RCTs included in our study was generally low. Second, despite the rigorous search strategy adopted and comprehensive reporting of our review on website, potential publication bias is very likely to exist. Third, the heterogeneity of the primary outcome in comparison of RT plus AT vs. AT is large which may to some extent affect the accuracy of the results. However, all of the outcomes are stable, and we chose a random effects model for the observed high heterogeneity. Meanwhile, we have try our best to explore the sources of heterogeneity by sensitivity and subgroup analysis.

### Implications for Clinical Practice

According to the analysis of the included trials, RT may be more suitable for the stable period of CHD. The application of exercise intervention needs clinical assessment to ensure safety (73). Although the current review (20) suggest that high-intensity resistance training may be more beneficial, we couldn't find correlation between the exercise intensity or intervention duration and favorable outcomes. According to animal experiments (74), RT may reduce oxidative stress through inflammatory factors such as TNF-α, thereby preventing endothelial dysfunction and atherosclerosis and reducing the risk of CVD (75). Our study suggests that RT is effective in improving QOL and LVEF, reducing LVEDD and the readmission rate compared with UC. Furthermore, there is no significant difference in the safety between RT and AT. Although more powerful evidence is required, it provides a potential for supplementing individual RT intervention for CHD rehabilitation in future clinical practices.

### Implications for Future Research

Future studies should be conducted more rigorously and follow the CONSORT statement. Clinical trials should be registered on the clinical trial websites and provide standardized trial protocols. Besides, exercise prescriptions require precise intensity and duration control, so more researches on different levels of intervention duration and intensity are necessary. In addition, clinical evidence and the analysis in this study confirm the effectiveness of RT in combination with AT, but large, high-quality clinical trials are still needed to confirm the effect of RT alone in patients with CHD. On the other hand, the outcome indicators of current trials focus on cardiopulmonary exercise function and muscle strength, other indicators affecting the prognosis of CHD patients, such as emotional condition (22) or flexibility (76), should also be taken into consideration. Since there is a certain heterogeneity among these studies in our review, comprehensive and standardized outcome indicators is essential to better evaluate the efficacy in future studies. Finally, long-term follow-up in future trials is conducive to better observation of the long-term efficacy and safety of RT in CHD.

## Conclusion

In conclusion, RT combined with AT is more beneficial than AT alone for CHD. RT can effectively improve the abilities of exercise and the quality of life in patients with CHD compared with UC. However, whether a difference existed between RT and AT is still unknown, and more high-quality and large-sample studies are warranted.

## Data Availability Statement

The original contributions presented in the study are included in the article/Supplementary Material, further inquiries can be directed to the corresponding author/s.

## Author Contributions

YF, MY, and HX: conceptualization and writing—review and editing. YF, MY, and TW: data curation. YF, HZ, and LZ: formal analysis. MY, JL, and QL: methodology. HX: project administration and supervision. YF, MY, JL, and HZ: writing—original draft. All authors contributed to the article and approved the submitted version.

## Funding

This work was supported by National Key R&D Program of China (No. 2018YFC2002502), CACMS Innovation Fund (CI2021A00917), and Beijing Traditional Chinese Medicine Science and Technology Development Fund program (No. JJ-2020-16).

## Conflict of Interest

The authors declare that the research was conducted in the absence of any commercial or financial relationships that could be construed as a potential conflict of interest.

## Publisher's Note

All claims expressed in this article are solely those of the authors and do not necessarily represent those of their affiliated organizations, or those of the publisher, the editors and the reviewers. Any product that may be evaluated in this article, or claim that may be made by its manufacturer, is not guaranteed or endorsed by the publisher.
