# Supplementary material for: Efficacy and Safety of Resistance Training for Coronary Heart Disease Rehabilitation: A Systematic Review of Randomized Controlled Trials
Source: Front Cardiovasc Med. 2021 Nov 5;8:754794. doi: 10.3389/fcvm.2021.754794 (PMC8602574; doi:10.3389/fcvm.2021.754794)
Supplement: Supplementary file 1 [file Data_Sheet_1.docx]

**Appendix 1. Search Strategy and Results**

We conducted a comprehensive search in four Chinese databases and three English databases from each individual database established to Dec,31,2020, and there was no language restriction. All this searching tasks run by Yixuan Fan and Meili Yu and exported to Note Express. The strategy searches for complementary as term and topic, and its alternate names as term. Resistance training and stable coronary heart disease context were included to narrow search results. To further narrow the search results, results were then combined with a search set for the term clinical trial. The details listed below.

| **Database** | **Website** | **Search Strategy** | **Outcome** | **Final Results** |
| --- | --- | --- | --- | --- |
| **Chinese National Knowledge Infrastructure** | https://www.cnki.net/ | #1(Title/Abstract/Keyword) “Kangzu” (resistance) OR “Kangzuxunlian” (resistance training) OR “Kangzuyundong” (resistance movement) OR “Zukangyundong” (resistance movement) OR “Kangzuli” OR “Kangzulianxi” (resistance exercise) OR “resistance training” OR “strength exercise” | 105872 | 753 |
|  |  | #2(Title/Abstract/Keyword) “Guanxinbing” (CHD) OR “Guanzhuangdongmaizhouyangyinghuaxingxinzangbing” (CHD) OR “Wuzhengzhuangxinjiquexue” (asymptomatic myocardial ischemia) OR “Yinnixingguanxinbing” (Occult coronary heart disease) OR “Quexuexingxinzangbing” (ischemic heart disease) OR “Wendingxingxinjiaotong” (stable angina) OR “Wendingxingguanxinbing” (stable coronary heart disease) OR “coronary heart disease” OR “CHD”  #3 #1 and #2 | 2889 |  |
|  |  | #4(Title/Abstract/Keyword) “Suiji” (random) OR “randomized controlled trial” OR “controlled clincial trial” OR “randomized” OR “placebo” OR “randomly” OR “trial” OR “randomised” OR “random”  #5 #3 and #4 | 753 |  |
| **VIP database** | http://lib.cqvip.com/ | (((((Title/Abstract) “Kangzu” (resistance) OR “Kangzuxunlian” (resistance training) OR “Kanzuyundong” (resistance movement) OR “Zukangyundong” (resistance movement) OR “Kangzuli” OR “Kangzulianxi” (resistance exercise) OR “resistance training” OR “strength exercise”)) AND ((Title/Abstract) “Guanxinbing” (CHD) OR “Guanzhuangdongmaizhouyangyinghuaxingxinzangbing” (CHD) OR “Wuzhengzhuangxinjiquexue” (asymptomatic myocardial ischemia) OR “Yinnixingguanxinbing” (Occult coronary heart disease) OR “Quexuexingxinzangbing” (ischemic heart disease) OR “Wendingxingxinjiaotong” (stable angina) OR “Wendingxingguanxinbing” (stable coronary heart disease) OR “coronary heart disease” OR “CHD”)) AND “All the journals”(Journal) AND “Medicine and health care”(Subjects) AND “The starting date” to 2020 (Date)) | 39 | 39 |
| **Chinese Biomedical Literature Service System** | http://www.sinomed.ac.cn/ | #1 (Full Text) “Kangzu” (resistance) OR “Kangzuxunlian” (resistance training) OR “Kangzuyundong” (resistance movement) OR “Zukangyundong” (resistance movement) OR “Kangzuli” OR “Kangzulianxi” (resistance exercise) OR “resistance training” OR “strength exercise” | 17046 | 79 |
|  |  | #2 (Full Text) “Guanxinbing” (CHD) OR “Guanzhuangdongmaizhouyangyinghuaxingxinzangbing” (CHD) OR “Wuzhengzhuangxinjiquexue” (asymptomatic myocardial ischemia) OR “Yinnixingguanxinbing” (Occult coronary heart disease) OR “Quexuexingxinzangbing” (ischemic heart disease) OR “Wendingxingxinjiaotong” (stable angina) OR “Wendingxingguanxinbing” (stable coronary heart disease) OR “coronary heart disease” OR “CHD” | 799331 |  |
|  |  | #3 (Full Text)“Suiji” (random) OR “randomized controlled trial” OR “controlled clincial trial” OR “randomized” OR “placebo” OR “randomly” OR “trial” OR “randomised” OR “random” | 2922997 |  |
|  |  | #4 #3 and #2 and #1 | 79 |  |
| **Wanfang database** | https://www.wanfangdata.com.cn/index.html | (((((Title/Abstract) “Kanzu” (resistance) OR “Kangzuxunlian” (resistance training) OR “Kanzuyundong” (resistance movement) OR “Zukangyundong” (resistance movement) OR “Kangzuli” OR “Kangzulianxi” (resistance exercise) OR “resistance training” OR “strength exercise”)) AND ((Title/Abstract) “Guanxinbing” (CHD) OR “Guanzhuangdongmaizhouyangyinghuaxingxinzangbing” (CHD) OR “Wuzhengzhuangxinjiquexue” (asymptomatic myocardial ischemia) OR “Yinnixingguanxinbing” (Occult coronary heart disease) OR “Quexuexingxinzangbing” (ischemic heart disease) OR “Wendingxingxinjiaotong” (stable angina) OR “Wendingxingguanxinbing” (stable coronary heart disease) OR “coronary heart disease” OR “CHD”)) AND (Topic) “Suiji” (random) OR “randomized controlled trial” OR “controlled clinical trial” OR “randomized” OR “placebo” OR “randomly” OR “trial” OR “randomised” OR “random”))*Date:-2020)) | 90 | 90 |
| **PubMed** | https://pubmed.ilibs.cn/ | #1 ((((((((((“resistance movement”[Title/Abstract]) OR “resistance training”[Title/Abstract]) OR “resistance exercise”[Title/Abstract]) OR “strength exercise”[Title/Abstract])) | 17324 | 52 |
|  |  | #2 ((((“coronary heart disease”[Title/Abstract]) OR “CHD”[Title/Abstract]) OR “ischemic heart disease”[Title/Abstract]) OR “coronary artery disease”[Title/Abstract]) OR “asymptomatic myocardial ischemia”[Title/Abstract]) OR “latent coronary heart disease”[Title/Abstract]) OR “ischemic heart failure”[Title/Abstract]) OR “ischemic heart disease”[Title/Abstract]) OR “coronary atherosclerotic cardiopathy”[Title/Abstract]) | 172017 |  |
|  |  | #3 #1 and #2 | 176 |  |
|  |  | #4 #3 and Filters: Clinical Trial, Randomized Controlled Trial | 52 |  |
| **Cochrane** **Library** | https://www.cochranelibrary.com/ | [Title/Abstract/Keyword]“resistance movement” OR “resistance training” OR “strength training” OR “resistance exercise” OR “strength exercise” AND [Title/Abstract/Keyword]“coronary heart disease” OR “CHD” OR “coronary atherosclerotic cardiopathy” OR “coronary artery disease” OR “asymptomatic myocardial ischemia” OR “latent coronary heart disease” OR “ischemic heart failure” OR “ischemic heart disease” OR “stable coronary heart disease” | 168 | 168 |
| **Embase** | https://www.embase.com/ | #1 (Title/Abstract/Keyword) “resistance movement” OR “resistance training” OR “strength training” OR “resistance exercise” OR “strength exercise” | 21725 | 121 |
|  |  | #2 (Title/Abstract/Keyword) “coronary heart disease” OR “CHD” OR “coronary atherosclerotic cardiopathy” OR “coronary artery disease” OR “asymptomatic myocardial ischemia” OR “latent coronary heart disease” OR “ischemic heart failure” OR “ischemic heart disease” OR “stable coronary heart disease” | 130270 |  |
|  |  | #3 #1 and #2 | 125 |  |
|  |  | #4 #3 and AND (“clinical article” OR “clinical study” OR “clinical trial” OR “cohort analysis” OR “comparative study” OR “control group” OR “controlled clinical trial” OR “controlled study” OR “crossover procedure” OR “double blind procedure” OR “evidence based practice” OR “experimental study” OR “human” OR “human experiment” OR “human tissue” OR “intermethod comparison” OR “intervention study” OR “major clinical study” OR “multicenter study” OR “multicenter study topic” OR “observational study” OR “outcomes research” OR “prospective study” OR “quantitative study” OR “questionnaire” OR “randomized controlled trial” OR “randomized controlled trial topic” OR “telephone interview”) | 121 |  |
