# Supplementary material for: Efficacy and Safety of Resistance Training for Coronary Heart Disease Rehabilitation: A Systematic Review of Randomized Controlled Trials
Source: Front Cardiovasc Med. 2021 Nov 5;8:754794. doi: 10.3389/fcvm.2021.754794 (PMC8602574; doi:10.3389/fcvm.2021.754794)
Supplement: Supplementary file 2 [file Data_Sheet_2.docx]

| **Appendix 2. Characteristics of 38 included trials** | | | | | | | | | | | | | | | | |
| --- | --- | --- | --- | --- | --- | --- | --- | --- | --- | --- | --- | --- | --- | --- | --- | --- |
| Included trials | Register | Country/Region | Diseases/population | Sample Size(T/C) | Age (years) | No. of Male/Female | Disease duration | Study desgin(T/C) | Interventions of resistance training | | | | Intervention duration | Outcome index | Follow-up duration | Adverse events |
|  |  |  |  |  |  |  |  |  | Mode | Treatment duration | Frequency (times/week) | Intensity(% of 1RM) |  |  |  |  |
| B Dwiputra 2016[30] | NCT02674659 | Indonesia | CABG | 44/43 | T:56.3±8.1  C:57.2±8.4 | 77/10 | None reported | RT+AT vs AT | Lower and upper extremities exercises | 3-4 weeks(12 times) | 3 or 5 | None | 3-4 weeks(12 times) | No indicators fit the bill | 2 months | T: acute heart failure (1); C: acute heart failure (1), wound infection (2) |
| B Yael 1999[31] | none | America | MI, CABG, coronary angioplasty, or new onset angina | 18/16 | T:58±12  C:59±12 | 25/9 | T:2.7±2.1  C:3.1±2.0  (months) | high-intensity strength training+AT vs flexibility training+AT | chest press, latissimus dorsi pull down, leg press, and knee extension, alternating upper and lower body exercises | 12 weeks | 2 | 50%(first week); 80%(second week); increased by 5% at the next session(12 repetitions could be performed in the third set) | 12 weeks | Skeletal muscle strength: chest press; knee extension | None | C: arthritic knee pain (1) |
| C Hung 2004[32] | none | Canada | MI | 9/9 | T:71±7  C:70±6 | 0/18 | None reported | RT+AT vs AT | chest press, shoulder press, vertical row, triceps extension, biceps curl, latissimus dorsi pull down, leg extension, and leg curl | 8 weeks | 3 | 55%; increased by 2.5% every week. | 8 weeks | 1. Skeletal muscle strength: shoulder press; biceps curl; chest press 2. QOL: physical; emotional; global | None | None reported |
| CH Luan 2019[33] | none | China | PCI | 50/50/50 | T1: 33.6±5.3 T2: 33.3±5.6 C: 33.2±6.3 | 111/39 | None reported | RT+AT vs AT vs UC(daily routines) | Stretch band | 1 year | 3(the first three months);2-3 | <30%(the first three months); 30%-50% | 1 year | 1.Peak VO_2_ 2.Anaerobic threshold  3.QOL: global | None | T1: rehospitalization for heart failure (1);  T2: percutaneous transluminal coronary intervention again (1), rehospitalization for heart failure (2); C: percutaneous transluminal coronary intervention again (3), myocardial infarction again (2), rehospitalization for heart failure (6) |
| D Hansen 2011[34] | ISRCTN81212339 | Belgium | AMI;Angina pectoris | 22/25 | T:60.4±8.9  C:58.9±7.2 | 44/3 | 18 (standard deviation 7) days  after coronary revascularization | RT+AT vs AT | sit leg extension, leg press | 6 weeks | 3 | 65% | 6 weeks | Peak VO_2_ | None | None reported |
| FR Caruso 2017[35] | RBR-63kf95 | America | MI; MRS | 10/10 | T:61.3±5.2 C:61±4.4 | 20/0 | T:3.3±0.9 C:3.5±0.8 (years) | RT+AT vs AT | High repetition/ low load resistance training(a leg press and  lower limb stretching),2 seconds of extension and 3 seconds of knee and hip flexion | 8 weeks | 2 | 30% | 8 weeks | Peak VO_2_ | 8 weeks | T: joint pain (1); C: joint pain (1) |
| H Dor-Haim 2018[36] | NCT01912690 | Israel | MI | 14/15 | 47–69 | 29/0 | 6–10 weeks post hospitalization due to acute MI | RT+AT vs AT | horizontal rowing, chest press, leg press, shoulder press, leg extension, lateral pull down, leg flexion and assisted squat | 12 weeks | none reported | 30%(in the first two weeks),increased to 50% | 12 weeks | QOL: physical; emotional | none | none |
| H Farheen 2018[37] | none | Pakistan | stable post-MI | 13/13 | T:57.23±9.76 C:55.77±10.46 | 16/10 | None reported | RT+AT vs AT | 1-3 kg of quadriceps resistive exercises | 6 weeks | 3 | 30%-50% | 6 weeks | LVEF | 6 weeks | None reported |
| HJ Jia 2018[62] | none | China | AMI,PCI | 58/58 | T:53.64±6.18 C:53.70±6.21 | 63/53 | None reported | RT vs UC(daily routines) | quadriceps stretches, calf lifts, shoulder lifts, chest pushes, triceps stretches and quadriceps flexes | 3 months | none reported | 50%;60%(after 8 weeks) | 3 months | 1.QOL: physical; emotional  2.LVEF;LVEDD | None | T: restenosis(4), angina(5); C: restenosis(10), angina(12), sinus arrhythmia(4), myocardial Infarction attack again(2) |
| HM Arthur 2007[38] | none | Canada | CABG,MI | 37/35 | none | 0/72 | None reported | RT+AT vs AT | leg press, knee extension, elbow  flexion, and seated upright bench press | 6 months | 2 | lower extremity:50%,70% by  4 weeks; upper extremity: 30%,70% of by 4 weeks | 6 months | 1.Peak VO_2_ 2.Skeletal muscle strength: arm flexion; leg flexion 3.QOL: physical; emotional | 1 year | None reported |
| J Li 2018[39] | none | China | CAD | 38/38 | T:55.6±1.6  C:55.7±1.5 | 41/35 | None reported | RT+AT vs AT | muscle group training:calf lifts, chest expansion exercises, latissimus dorsi pulldown, triceps stretch, etc | 3 months | none reported | None | 3 months | QOL: global | None | None reported |
| J-P Schmid 2008[40] | none | Switzerland | MI | 17/21 | T:54.7±9.4  C:57.0±9.6 | 32/8 | 4-12 weeks | RT+AT vs AT(endurance exercise) | leg press, leg curl, back extension, abdom inal crunch, abdominal oblique crunch and latissimus pull | 3 months | 6 | 40%-60%(in 4-week intervals) | 3 months | 1.Peak VO_2_ 2.LVEF | 9 months | None reported |
| KQ Wu 2017[63] | none | China | AMI,PCI | 30/34 | 60-80 | 53/11 | None reported | RT vs UC(drug treatment and daily routines) | stretch band resistance training | 6 months | 3 | none | 6 months | No indicators fit the bill | None | T: chest discomfort(22); C: chest discomfort(29) |
| LA Coke 2008[41] | none | America | AMI,PCI,CABG,stable angina | 16/16 | T:64±11  C:65±10 | 0/32 | None reported | RT+AT vs AT | chest press, shoulder press, biceps curl, lateral row left and right, and tri ceps extension left and right | 10 weeks | 2 | 40%,50%(weeks 4-6),60%(weeks 7-10) | 12 weeks | Skeletal muscle strength: shoulder press; biceps curl; chest press | None | None reported |
| LD Zhai 2018[42] | none | China | SCAD | 20/19/18 | T1: 60.8±5.6 T2:60.0±6.7 C: 61.9±5.7 | 18/39 | T1: 4.5  T2:5.3 C: 3.9 (years) | RT+AT vs AT vs UC(drug treatment) | strength training of upper limbs, waist and abdomen, lower back and lower limb muscle groups | 12 weeks | 3 | None | 12 weeks | 1.Peak VO_2_ 2.QOL: physical; emotional | None | none |
| LW Luo 2020[64] | none | China | PCI | 20/20 | <50 | None | None reported | RT vs UC(drug treatment) | resistance band training | 1 year | 3 | ＜30%,40% | 1 year | LVEF | None | None reported |
| M Gayda 2009[43] | none | Canada | MI,CABS,Angioplasty | 8/8 | 55±8 | 16/0 | None reported | RT+AT vs AT | quadriceps leg extension and lateral pull down | 7 weeks | 3 | 40% | 7 weeks | Peak VO_2_ | None | None reported |
| M Vona 2009[44] | none | Switzerland | AMI,PTCA | 53/54/52/50 | T1: 55±9 T2: 57±8 T3: 56±6 C: 58±7 | 155/54 | None reported | RT+AT vs RT vs AT vs UC(no training) | weights and rubber bands | 1 months | T1:2;T2:4 | 60% | 1 months | Peak VO_2_ | 1 month | none |
| MD Guan 2013[45] | none | China | STEMI,PCI | 32/30 | T:60.1±10.7  C:61.2±11.1 | 36/26 | None reported | RT+AT vs AT | leg extension, latissimus latissimus pull-up, chest push, butterfly chest expansion, bicep push, neck arm flexure and extension, push up rowing and latissimus latissimus pull-up | 6 months | 3 | 50%(the first month),60%(the second month) | 6 months | 1.QOL: global  2.LVEDD | 12 months | None reported |
| MH Kelemen1986[46] | none | America | MI, CABG, angina | 20/20 | 55±8.5 | 40/0 | None reported | RT+AT vs AT | arms and upper torso: vertical fly, arm curl, shoulder press,high pulley,low pulley,bench press; legs:double leg curl,double leg extension; abdomen/hips (no weight measurements):bent leg sit-ups,hip flexor. | 10 weeks | 3 | 40% | 10 weeks | Skeletal muscle strength: shoulder press; arm flexion; leg flexion; knee extension | None | T: symptomatic hypotension and responded to the oral administration of fluids (1), brief episodes of ventricular bigeminy (4), isolated premature ventricular complexes (4) |
| PL M 2001[47] | none | America | MI, CABG, angioplasty | 10/10 | T:58.7±8.3  C:61±8 | 13/7 | None reported | RT+AT vs AT | knee extension, hamstring curl, seated chest press, pull-over, shoulder press, bicep curl, and tricep extension,forearm curls with free weight dumbbells and hand-grip exercise with spring-loaded grippers | 6 months | 3 | 40%(increased as tolerated thereafter) | 6 months | 1.Peak VO_2_  2.Skeletal muscle strength: shoulder press; biceps curl; chest press; leg flexion; knee extension | None | T: low back pain (4), elbow tendonitis (1), shoulder pain (1) |
| PM Leprêtre 2016[48] | none | France | coronary angioplasty | 16/16 | T:63.1±7  C:64.6±9.1 | 32/0 | None reported | RT+AT vs AT | lower limb exercises (leg press, quadriceps leg extension and standing calf raise) and upper limb exercises (lateral raise, triceps pushdown and dumbbell curl) | 4 weeks | 5 | 30%-50% | 4 weeks | Peak VO_2_ | None | None reported |
| Q Liang 2020[49] | none | China | PCI | 40/40 | T:65.03±9.96  C:63.35±9.92 | 58/22 | None reported | RT+AT vs AT | resistance band training | 12 weeks | 3 | <30%; 30%-50% | 12 weeks | 1.VO_2_ max 2.Anaerobic threshold 3.QOL: physical; emotional  4.LVEF | None | None reported |
| RJ Wang 2013[50] | none | China | CHD | 36/36 | Male:66.5±4.6  Female:61.2±4.8 | 34/38 | None reported | RT+AT vs AT | diagonal brace, heel lift, lower limb loading side kick, front kick, half squat with upper limb assistance;comprehensive strength exercises on waist and abdominal muscles, elbow flexion and extension, knee flexion and extension, and thigh adduction and abduction | 12 weeks | 3 | 15-20 | 12 weeks | 1.Skeletal muscle strength: arm flexion;leg flexion; knee extension 2.QOL: physical; emotional; global | None | None reported |
| S Ghroubi 2013[60] | none | Tunisia | CABG,MI | 16/16 | T:59.25±1.7  C:59±5.85 | none | T:73.1±14  C:60±26 (days from CABG) | RT vs AT | muscle strength training:low-resistance of the lower limbs,knee extension and flexion | 8 weeks | 3 | None | 8 weeks | 1.Peak VO_2_ 2.QOL: physical; emotional | None | T: knee pain (3); C: knee pain (5), significant exercise-induced ST-segment depression without chest pain (2) |
| S Marzolini 2008[51] | none | Canada | CABG,PCI,MI,CAD(no MI or intervention) | 19/18/16 | T1:60.9 ± 2.3 T2: 62.7 ± 2.7 C:57.9 ± 2.6 | 47/6 | None reported | AT+RT1 vs AT+RT3 vs AT | three specific to the lower body (2 with dumbbells one with Theraband exercise bands), 5 upper body (dumbbells) and two trunk-stabilizing exercises (patient's body weight) | 24 weeks | 6 | 60%;70%-75% | 24 weeks | Peak VO_2_ | None | none |
| S Marzolini 2015[52] | none | Canada | CABG,PCI,MI,CAD(no MI or intervention) | 19/18/16 | T1:60.9 ± 9.9 T2: 62.7 ± 11,4 C:57.9 ± 10.5 | 47/6 | None reported | AT+RT1 vs AT+RT3 vs AT | three specific to the lower body (2 with dumbbells one with Theraband exercise bands), 5 upper body (dumbbells) and two trunk-stabilizing exercises (patient's body weight) | 24 weeks | 6 | 60%;70%-75% | 24 weeks | QOL: physical; emotional | None | none |
| SH Zhang 2018[65] | none | China | PCI | 33/34 | T:62.07±9.22  C:61.3±9.07 | 35/32 | PCI surgery was performed within 3 months | RT vs usual care(daily routines and drug treatment) | Low intensity regular resistance movement (MOTOmed viva2 lower extremity sports rehabilitation apparatus) | 12 weeks | 5 | None | 12 weeks | No indicators fit the bill | 3 months,6months | T: angina (8), myocardial Infarction (1), ventricular arrhythmias (3); rehospitalization (4) C: angina(17), myocardial Infarction (3), ventricular arrhythmias (9), heart failure (3); rehospitalization (15) |
| SJ Tan 2007[53] | none | China | CAD | 22/22 | 61.3±5 | 21/23 | None reported | RT+AT vs AT | Lumbago and abdominal muscles, upper arm muscles, leg muscles and other combination of strength exercises | 12 weeks | 3 | 50%-60% | 12 weeks | 1.Peak VO_2_ 2.Skeletal muscle strength: arm flexion; leg flexion; knee extension 3.LVEF | None | None |
| XH Liu 2018[54] | none | China | SCAD | 35/35/35 | T1:61.13 ± 8.73 T2: 59.84 ± 7.98 C:60.67 ± 8.44 | 76/29 | None reported | RT+AT vs AT vs UC(daily routine) | resistance band training | 12 weeks | 2 | Upper limb:30%-40%;  Lower limbs:50%-60% | 12 weeks | 1.peak VO_2_ 2.Skeletal muscle strength:arm flexion;leg flexion; knee extension 3.Anaerobic threshold | None | None |
| XW Zheng 2019[55] | none | China | AMI,unstable angina,PCI | 46/46 | T:59.82±10.02 C:60.25±10.21 | 53/39 | None reported | RT+AT vs AT | stretch band progressive resistance training | 12 weeks | 2-3 | <30%;30%-50% | 12 weeks | 1.Peak VO_2_ 2.QOL: physical; emotional 3.Anaerobic threshold 4. LVEF; LVEDD | None | None |
| XY Gu 2003[56] | none | China | SCAD | 20/20 | T:60±9 C:60±8 | none | None reported | RT+AT vs AT | (hand held dumbbell)(left and right neck rotation, forward tiptoe, front lunge, side tiptoe, front pile, side lunge, side pile) | 6 weeks | 5 | 70％-85％HRmax | 6 weeks | LVEF; LVEDD | None | None |
| Y Du 2015[61] | none | China | PCI | 100/100 | T:64.6±3.2 C:66.8±4.6 | 79/121 | None reported | RT vs AT | stretch band progressive resistance training | 1 year | 3 | <30%;30%-50% | 1 year | 1.PeakVO_2_  2.QOL: physical; emotional 3.Anaerobic threshold 4.LVEF | None | None |
| Y Tang 2019[57] | none | China | AMI,PCI | 30/30/30 | T1:67.25±3.15 T2: 68.23±2.13 C:68.35±3.03 | 57/33 | None reported | RT+AT vs AT vs UC(drug treatment) | chest and back muscle training, lumbar and abdominal muscle training, hip and leg muscle training | none | 3 | Chest and back muscle training:30%-40%; Lumbar and abdominal muscle training, hip and leg muscle training:50%-60% | none | LVEF | None | None |
| YH Gao 2019[65] | none | China | CAD | 40/40 | T:75.83±8.02 C:78.7±6.76 | 39/41 | T:19.25±4.94  C:19.18±4.06 (years) | RT vs UC(daily routines) | stretch band progressive resistance training | 6 months | 3 | none | 6 months | LVEF; LVEDD | None | T: rehospitalization (6);  C: rehospitalization (16) |
| YY Ouyang 2017[58] | none | China | PCI | 19/19/16 | 20/20/20: T1:54.3±10.8 T2:53.2±11.7 C:51.2±11.4 | 45/15 | None reported | RT+AT vs AT vs UC(daily routines) | lumbar back, abdominal muscle group and upper and lower limb muscle group exercise | 12 months | 3 | 50%,60% | 12 months | 1.VO_2_ max 2.QOL:global 3.LVEF | None | None |
| YY Su 2018[67] | none | China | SCAD | 45/47 | T:64.36±4.95 C:63.47±4.48 | 40/52 | None reported | RT vs UC(daily routines) | stretch band progressive resistance training | 3 months | 3 times a week or every other day | Upper limb:30%-40%;  Lower limbs:50%-60%; (No more than 50% ~ 70%) | 3 months | QOL: physical; emotional | None | None |
| Z Khalid 2019[59] | none | Pakistan | AMI | 13/13 | T:57.23±9.757 C:55.77±10.457 | 16/10 | None reported | RT+AT vs AT | bilateral hand grippers and biceps curls lifting;quads  resistive exercises on quadriceps chair;walking/uphill running on treadmill  standing calf raise exercise;a cool down period | 6 weeks | 3 | 60%-85% | 6 weeks | QOL: physical; emotional | None | None |
| Abbreviation: T: treatment group; C: control group; RT: resistance training; AT: aerobic training; UC: usual care; 1 RM: 1 Repitions Maximum.  Note: Diseases: CABG: coronary artery bypass grafting; MI: myocardial infarction; PCI: percutaneous transluminal coronary intervention; AMI: acute myocardial infarction; MRS:myocardial revascularization surgery; CAD: coronary artery disease; SCAD: stable coronary artery disease; CABS: coronary artery bypass surgery; PTCA: percutaneous transluminal coronary angioplasty; STEMI: ST-segment elevation myocardial infarction; CHD: coronary heart disease. Outcomes: Peak VO_2_: peak oxygen uptake; VO_2_ max: maximum oxygen uptake; QOL: quality of life; LVEF: left ventricular ejection fraction; LVEDD: left ventricular end-diastolic dimension | | | | | | | | | | | | | | | | |
