# Supplementary material for: Efficacy and Safety of Resistance Training for Coronary Heart Disease Rehabilitation: A Systematic Review of Randomized Controlled Trials
Source: Front Cardiovasc Med. 2021 Nov 5;8:754794. doi: 10.3389/fcvm.2021.754794 (PMC8602574; doi:10.3389/fcvm.2021.754794)
Supplement: Supplementary file 3 [file Data_Sheet_3.docx]

| **Appendix 3. Cochrane Risk of Bias Assessment** | | | | | | | |
| --- | --- | --- | --- | --- | --- | --- | --- |
| Criterion  Study ID | Random Sequence generation | Allocation concealment | Blinding of participants and personnel | Blinding of outcome assessment | Incomplete outcome data | Selective outcome reporting | Other potential risk of bias |
| B Dwiputra 2016[30] | U | U | L | U | L | L | L |
| B Yael 1999[31] | U | U | L | L | L | L | L |
| C Hung 2004[32] | U | U | U | U | L | L | U |
| CH Luan 2019[33] | L | U | U | U | L | L | U |
| D Hansen 2011[34] | L | U | U | L | L | L | L |
| FR Caruso 2017[35] | U | L | L | L | L | L | L |
| H Dor-Haim 2018[36] | U | L | L | L | L | L | L |
| H Farheen 2018[37] | L | U | U | U | L | L | U |
| HJ Jia 2018[62] | U | U | U | U | L | L | U |
| HM Arthur 2007[38] | L | L | L | L | L | L | L |
| J Li 2018[39] | U | U | U | U | L | L | U |
| J-P Schmid 2008[40] | U | U | U | U | L | L | U |
| KQ Wu 2017[63] | U | U | U | U | L | L | L |
| LA Coke 2008[41] | U | U | U | U | L | L | L |
| LD Zhai 2018[42] | U | U | U | U | L | L | U |
| LW Luo 2020[64] | U | U | U | U | L | L | U |
| M Gayda 2009[43] | U | U | U | U | L | L | L |
| M Vona 2009[44] | U | U | L | L | L | L | U |
| MD Guan 2013[45] | U | U | U | U | L | L | U |
| MH Kelemen1986[46] | U | U | U | U | L | L | U |
| PL M 2001[47] | U | U | U | U | L | L | L |
| PM Leprêtre 2016[48] | U | U | L | U | L | L | L |
| Q Liang 2020[49] | L | U | U | U | L | L | L |
| RJ Wang 2013[50] | L | U | U | U | L | L | U |
| S Ghroubi 2013[60] | U | U | U | U | L | L | L |
| S Marzolini 2008[51] | L | L | L | L | L | L | L |
| S Marzolini 2015[52] | L | L | L | L | L | L | L |
| SH Zhang 2018[65] | L | U | L | L | L | L | L |
| SJ Tan 2007[53] | U | U | U | U | L | L | U |
| XH Liu 2018[54] | U | U | U | U | L | L | U |
| XW Zheng 2019[55] | U | L | U | U | L | L | U |
| XY Gu 2003[56] | U | U | U | U | L | L | L |
| Y Du 2015[61] | U | U | L | L | L | L | U |
| Y Tang 2019[57] | U | U | U | L | L | L | U |
| YH Gao 2019[65] | L | U | U | U | L | L | U |
| YY Ouyang 2017[58] | L | U | U | U | L | L | L |
| YY Su 2018[67] | L | L | U | U | L | L | U |
| Z Khalid 2019[59] | L | U | L | U | L | L | L |
