# Supplementary material for: Efficacy and Safety of Resistance Training for Coronary Heart Disease Rehabilitation: A Systematic Review of Randomized Controlled Trials
Source: Front Cardiovasc Med. 2021 Nov 5;8:754794. doi: 10.3389/fcvm.2021.754794 (PMC8602574; doi:10.3389/fcvm.2021.754794)
Supplement: Supplementary file 4 [file Data_Sheet_4.docx]

| **Appendix 4. Estimate the efficacy and safety of RT for the rehabilitation in CHD** | | | | | |
| --- | --- | --- | --- | --- | --- |
| Outcome or subgroup title | | No. of studies | No. of participants | Effect estimate (95%CI) | P value* |
| **1. Peak VO_2_ and VO_2_ max** | | | | | |
| **1.1** **RT+AT versus AT** | | | | | |
| ***RT+AT versus AT (Peak VO_2_)*** | | | | | |
|  | CH Luan 2019 | 1 | 100 | MD 2.95 ( 1.60 to 4.30) | P<0.0001 |
|  | D Hansen 2011 | 1 | 47 | MD -0.7 ( -4.54 to 3.14) | P=0.72 |
|  | FR Caruso 2017 | 1 | 20 | MD 2.80 ( -1.36 to 6.96) | P=0.19 |
|  | HM Arthur 2007 | 1 | 72 | MD -0.94 ( -2.63 to 0.75) | P=0.28 |
|  | J-P Schmid 2008 | 1 | 38 | MD 1.40 ( -2.58 to 5.38) | P=0.49 |
|  | LD Zhai 2018 | 1 | 39 | MD 3.18 ( 0.31 to 6.05) | P=0.03 |
|  | M Gayda 2009 | 1 | 16 | MD 7.00 ( 2.21 to 11.79) | P=0.004 |
|  | M Vona 2009 | 1 | 105 | MD 0.50 ( -0.11 to 1.11) | P=0.11 |
|  | PL M 2001 | 1 | 20 | MD 6.90 ( 2.38 to 11.42) | P=0.003 |
|  | PM Leprêtre 2016 | 1 | 32 | MD 1.30 ( -1.45 to 4.05) | P=0.35 |
|  | S Marzolini 2008 | 1 | 35 | MD 0.00 ( -0.83 to 0.83) | P=1.00 |
|  | S Marzolini 2008(RT3+AT) | 1 | 34 | MD -1.10 ( -1.91 to -0.29) | P=0.008 |
|  | SJ Tan 2007 | 1 | 44 | MD -0.39 ( -1.66 to 0.88) | P=0.55 |
|  | XH Liu 2018 | 1 | 70 | MD 2.20 ( 0.35 to 4.05) | P=0.02 |
|  | XW Zheng 2019 | 1 | 92 | MD 3.77 ( 2.14 to 5.40) | P<0.00001 |
|  | ***Subtotal*** | 14 | 764 | **MD 1.36 ( 0.40 to 2.31)** | **P=0.005** |
| ***RT+AT versus AT (VO_2_ max)*** | | | | | |
|  | Q Liang 2020 | 1 | 80 | MD 1.52 ( 0.52 to 2.52) | P=0.003 |
|  | YY Ouyang 2017 | 1 | 38 | MD -0.80( -4.31 to 2.71) | P=0.66 |
|  | ***Subtotal*** | 2 | 118 | **MD 1.26 ( 0.41 to 2.12)** | **P=0.30** |
| **1.2 RT versus AT (Peak VO_2_)** | | | | | |
|  | M Vona 2009 | 1 | 106 | MD 0.20 ( -0.37 to 0.77) | P=0.49 |
|  | S Ghroubi 2013 | 1 | 32 | MD 0.40 ( -3.13 to 3.93) | P=0.82 |
|  | Y Du 2015 | 1 | 200 | MD 5.30 ( 4.23 to 6.37) | P<0.00001 |
|  | ***Subtotal*** | 4 | 338 | **MD 2.07 (-1.96 to 6.09)** | **P=0.31** |
| **1.3 RT versus UC (Peak VO_2_)** | | | | | |
|  | M Vona 2009 | 1 | 104 | MD 3.10 ( 2.52 to 3.68) | P<0.00001 |
|  | ***Subtotal*** | 2 | 104 | **MD 3.10 ( 2.52 to 3.68)** | **P<0.00001** |
| **2. QOL** | | | | | |
| *Subgroup 1: Physical score* | | | | | |
| **2.1.1 RT+AT versus AT** | | | | | |
|  | C Hung 2004 | 1 | 18 | SMD 0.54 (-0.41 to 1.48) | P=0.26 |
|  | H Dor-Haim 2018 | 1 | 29 | SMD 2.14 (1.20 to 3.07) | P<0.00001 |
|  | HM Arthur 2007 | 1 | 72 | SMD 0.65 (0.17 to 1.12) | P=0.007 |
|  | LD Zhai 2018 | 1 | 39 | SMD -0.10 (-0.72 to 0.53) | P=0.77 |
|  | Q Liang 2020 | 1 | 80 | SMD 0.71 (0.26 to 1.16) | P=0.002 |
|  | RJ Wang 2013 | 1 | 72 | SMD 1.80 (1.24 to 2.35) | P<0.00001 |
|  | S Marzolini 2015 | 1 | 35 | SMD 0.49 (-0.18 to 1.17) | P=0.15 |
|  | S Marzolini 2015(RT3+AT) | 1 | 34 | SMD 0.18 (-0.50 to 0.89) | P=0.61 |
|  | XW Zheng 2019 | 1 | 92 | SMD 0.50 (0.09 to 0.92) | P=0.02 |
|  | Z Khalid 2019 | 1 | 26 | SMD 0.37 (-0.41 to 1.14) | P=0.36 |
|  | ***Subtotal*** | 10 | 497 | **SMD 0.71 (0.33 to 1.08)** | **P=0.0003** |
| **2.1.2 RT versus AT** | | | | | |
|  | S Ghroubi 2013 | 1 | 32 | SMD 0.39 (-0.31 to 1.09) | P=0.27 |
|  | Y Du 2015 | 1 | 200 | SMD 0.14 (-0.14 to 0.42) | P=0.32 |
|  | ***Subtotal*** | 2 | 232 | **SMD 0.18 (-0.08 to 0.43)** | **P=0.18** |
| **2.1.3 RT versus UC** | | | | | |
|  | HJ Jia 2018 | 1 | 116 | SMD 0.79 (0.41 to 1.17) | P<0.0001 |
|  | YY Su 2018 | 1 | 92 | SMD 0.93 (0.50 to 1.36) | P<0.0001 |
|  | ***Subtotal*** | 2 | 208 | **SMD 0.85 (0.57 to 1.14)** | **P<0.00001** |
| *Subgroup 2: Emotional score* | | | | | |
| **2.2.1 RT+AT versus AT** | | | | | |
|  | C Hung 2004 | 1 | 18 | SMD 0.12 (-0.81 to 1.04) | P=0.80 |
|  | H Dor-Haim 2018 | 1 | 29 | SMD -1.12 (-1.91 to -0.33) | P=0.006 |
|  | HM Arthur 2007 | 1 | 72 | SMD 0.05 (-0.41 to 0.52) | P=0.82 |
|  | LD Zhai 2018 | 1 | 39 | SMD 0.38 (-0.26 to 1.01) | P=0.25 |
|  | Q Liang 2020 | 1 | 80 | SMD 0.92 (0.46 to 1.38) | P<0.0001 |
|  | RJ Wang 2013 | 1 | 72 | SMD 0.00 (-0.46 to 0.46) | P=1.00 |
|  | S Marzolini 2015 | 1 | 35 | SMD 0.11 (-0.55 to 0.78) | P=0.74 |
|  | S Marzolini 2015(RT3+AT) | 1 | 34 | SMD 0.12 (-0.55 to 0.80) | P=0.72 |
|  | XW Zheng 2019 | 1 | 92 | SMD 0.62 (0.20 to 1.04) | P=0.004 |
|  | Z Khalid 2019 | 1 | 26 | SMD 1.32 (0.45 to 2.18) | P=0.003 |
|  | ***Subtotal*** | 10 | 497 | **SMD 0.27 (-0.08 to 0.61)** | **P=0.13** |
| **2.2.2 RT versus AT** | | | | | |
|  | S Ghroubi 2013 | 1 | 32 | SMD 0.57 (-0.14 to 1.28) | P=0.12 |
|  | Y Du 2015 | 1 | 200 | SMD 0.12 (-0.16 to 0.40) | P=0.40 |
|  | ***Subtotal*** | 2 | 232 | **SMD 0.22 (-0.15 to 0.59)** | **P=0.24** |
| **2.2.3 RT versus UC** | | | | | |
|  | HJ Jia 2018 | 1 | 116 | SMD 0.96 (0.58 to 1.35) | P<0.00001 |
|  | YY Su 2018 | 1 | 92 | SMD 0.51 (0.10 to 0.93) | P=0.02 |
|  | ***Subtotal*** | 2 | 208 | **SMD 0.74 (0.31 to 1.18)** | **P=0.0009** |
| *Subgroup 3: Global score* | | | | | |
| **2.3.1 RT+AT versus AT** | | | | | |
|  | C Hung 2004 | 1 | 18 | SMD 0.36 (-0.57 to 1.29) | P=0.45 |
|  | CH Luan 2019 | 1 | 100 | SMD 0.62 (0.22 to 1.03) | P=0.002 |
|  | J Li 2018 | 1 | 76 | SMD 0.87 (0.40 to 1.35) | P=0.0003 |
|  | MD Guan 2013 | 1 | 62 | SMD 0.54 (0.04 to 1.05) | P=0.04 |
|  | RJ Wang 2013 | 1 | 72 | SMD 1.61 (1.08 to 2.15) | P<0.00001 |
|  | YY Ouyang 2017 | 1 | 38 | SMD 0.48 (-0.17 to 1.12) | P=0.15 |
|  | ***Subtotal*** | 6 | 366 | **SMD 0.78 (0.43 to 1.14)** | **P<0.0001** |
| **2.3.2 RT versus UC** | | | | | |
|  | YY Su 2018 | 1 | 92 | SMD 1.40 (0.94 to 1.85) | P<0.00001 |
|  | ***Subtotal*** | 1 | 92 | **SMD 1.40 (0.94 to 1.85)** | **P<0.00001** |
| **3.Skeletal muscle strength (RT+AT versus AT)** | | | | | |
| *Subgroup 1: Shoulder press* | | | | | |
|  | C Hung 2004 | 1 | 18 | SMD 0.57 (-0.38 to 1.52) | P=0.24 |
|  | LA Coke 2008 | 1 | 32 | SMD 1.23 (0.47 to 2.00) | P=0.002 |
|  | MH Kelemen1986 | 1 | 40 | SMD 0.00 (-0.62 to 0.62) | P=1.00 |
|  | PL M 2001 | 1 | 20 | SMD 1.56 (0.53 to 2.59) | P=0.003 |
|  | ***Subtotal*** | 4 | 110 | **SMD 0.79 (0.06 to 1.52)** | **P=0.03** |
| *Subgroup 2: Biceps curl* | | | | | |
|  | C Hung 2004 | 1 | 18 | SMD 0.71 (-0.25 to 1.68) | P=0.15 |
|  | LA Coke 2008 | 1 | 32 | SMD 1.31 (0.53 to 2.08) | P=0.0009 |
|  | PL M 2001 | 1 | 20 | SMD 0.74 (-0.18 to 1.65) | P=0.11 |
|  | ***Subtotal*** | 3 | 70 | **SMD 0.97 (0.47 to 1.48)** | **P=0.0001** |
| *Subgroup 3: Chest press* | | | | | |
|  | B Yael 1999 | 1 | 34 | SMD 0.64 (-0.05 to 1.33) | P=0.07 |
|  | C Hung 2004 | 1 | 18 | SMD 0.84 (-0.13 to 1.82) | P=0.09 |
|  | LA Coke 2008 | 1 | 32 | SMD 1.34 (0.57 to 2.12) | P=0.0007 |
|  | PL M 2001 | 1 | 20 | SMD 1.10 (0.05 to 2.06) | P=0.02 |
|  | ***Subtotal*** | 4 | 104 | **SMD 0.96 (0.55 to 1.37)** | **P<0.00001** |
| *Subgroup 4: Arm flexion* | | | | | |
|  | HM Arthur 2007 | 1 | 72 | SMD 0.49 (0.02 to 0.69) | P=0.04 |
|  | MH Kelemen1986 | 1 | 40 | SMD 0.71 (0.06 to 1.35) | P=0.03 |
|  | RJ Wang 2013 | 1 | 72 | SMD 0.45 (-0.02 to 0.91) | P=0.06 |
|  | SJ Tan 2007 | 1 | 44 | SMD 0.44 (-0.16 to 1.04) | P=0.15 |
|  | XH Liu 2018 | 1 | 70 | SMD 0.27 (-0.21 to 0.74) | P=0.27 |
|  | ***Subtotal*** | 5 | 298 | **SMD 0.45 (0.22 to 0.68)** | **P=0.0002** |
| *Subgroup 5: Leg flexion* | | | | | |
|  | HM Arthur 2007 | 1 | 72 | SMD 0.07 (-0.39 to 0.53) | P=0.77 |
|  | MH Kelemen1986 | 1 | 40 | SMD 0.21 (-0.42 to 0.83) | P=0.52 |
|  | PL M 2001 | 1 | 20 | SMD 1.46 (0.45 to 2.47) | P=0.005 |
|  | RJ Wang 2013 | 1 | 72 | SMD 1.58 (1.05 to 2.11) | P<0.00001 |
|  | SJ Tan 2007 | 1 | 44 | SMD 1.57 (0.89 to 2.25) | P<0.00001 |
|  | XH Liu 2018 | 1 | 70 | SMD 1.07 (0.56 to 1.57) | P<0.0001 |
|  | ***Subtotal*** | 6 | 318 | **SMD 0.96 (0.38 to 1.55)** | **P=0.001** |
| *Subgroup 6: Knee extension* | | | | | |
|  | B Yael 1999 | 1 | 34 | SMD 1.13 (0.40 to 1.86) | P=0.002 |
|  | MH Kelemen1986 | 1 | 40 | SMD 1.39 (0.69 to 2.09) | P<0.0001 |
|  | PL M 2001 | 1 | 20 | SMD 0.57 (-0.32 to 1.47) | P=0.21 |
|  | RJ Wang 2013 | 1 | 72 | SMD 0.99 (0.50 to 1.48) | P<0.0001 |
|  | SJ Tan 2007 | 1 | 44 | SMD 0.98 (0.35 to 1.61) | P=0.002 |
|  | XH Liu 2018 | 1 | 70 | SMD 0.53 (0.05 to 1.01) | P=0.03 |
|  | ***Subtotal*** | 6 | 280 | **SMD 0.90 (0.65 to 1.16)** | **P<0.00001** |
| **4.Anaerobic threshold** | | | | | |
| **4.1 RT+AT versus AT** | | | | | |
|  | CH Luan 2019 | 1 | 100 | MD 1.34 (0.61 to 2.07) | P=0.0003 |
|  | Q Liang 2020 | 1 | 80 | MD 1.18 (0.24 to 2.12) | P=0.01 |
|  | XH Liu 2018 | 1 | 70 | MD 1.30 (0.31 to 2.29) | P=0.010 |
|  | XW Zheng 2019 | 1 | 92 | MD 2.99 (1.77 to 4.21) | P<0.00001 |
|  | ***Subtotal*** | 4 | 342 | **MD 1.61 (0.91 to 2.31)** | **P<0.00001** |
| **4.2 RT versus AT** | | | | | |
|  | Y Du 2015 | 1 | 200 | MD 2.40 (1.50 to 3.30) | P<0.00001 |
|  | ***Subtotal*** | 1 | 200 | **MD 2.40 (1.50 to 3.30)** | **P<0.00001** |
| **5. Left ventricular function and structure** | | | | | |
| *Subgroup 1: LVEF* | | | | | |
| **5.1.1 RT+AT versus AT** | | | | | |
|  | CH Luan 2019 | 1 | 100 | MD 6.05 (3.41 to 8.69) | P<0.00001 |
|  | H Farheen 2018 | 1 | 26 | MD 5.00 (-1.08 to 11.08) | P=0.11 |
|  | J-P Schmid 2008 | 1 | 38 | MD -4.10 (-12.84 to 4.64) | P=0.36 |
|  | Q Liang 2020 | 1 | 80 | MD 3.68 (0.36 to 7.00) | P=0.03 |
|  | SJ Tan 2007 | 1 | 44 | MD 0.34 (-5.73 to 6.41) | P=0.91 |
|  | XW Zheng 2019 | 1 | 92 | MD 6.49 (2.12 to 10.86) | P=0.004 |
|  | XY Gu 2003 | 1 | 40 | MD -1.00 (-5.84 to 3.84) | P=0.69 |
|  | Y Tang 2019 | 1 | 60 | MD 7.07 (3.97 to 10.17) | P<0.00001 |
|  | YY Ouyang 2017 | 1 | 38 | MD 5.50 (0.94 to 10.06) | P=0.02 |
|  | ***Subtotal*** | 9 | 518 | **MD 4.17 (2.15 to 6.19)** | **P<0.0001** |
| **5.1.2 RT versus AT** | | | | | |
|  | Y Du 2015 | 1 | 200 | MD 4.60 (2.52 to 6.68) | P<0.0001 |
|  | ***Subtotal*** | 1 | 200 | **MD 4.60 (2.52 to 6.68)** | **P<0.0001** |
| **5.1.3 RT versus UC** | | | | | |
|  | HJ Jia 2018 | 1 | 116 | MD 6.64 (4.45 to 8.83) | P<0.00001 |
|  | LW Luo 2020 | 1 | 40 | MD 4.50 (1.40 to 7.60) | P=0.004 |
|  | YH Gao 2019 | 1 | 80 | MD 12.08 (8.73 to 15.43) | P<0.00001 |
|  | ***Subtotal*** | 3 | 236 | **MD 7.65 (3.73 to 11.57)** | **P=0.0001** |
| *Subgroup 2: LVEDD* | | | | | |
| **5.2.1 RT+AT versus AT** | | | | | |
|  | MD Guan 2013 | 1 | 62 | MD -1.20 (-3.17 to 0.77) | P=0.23 |
|  | XW Zheng 2019 | 1 | 92 | MD -12.01 (-15.56 to -8.46) | P<0.00001 |
|  | XY Gu 2003 | 1 | 40 | MD -0.45 (-4.69 to 3.79) | P=0.84 |
|  | ***Subtotal*** | 3 | 194 | **MD -4.53 (-11.55 to 2.48)** | **P=0.21** |
| **5.2.2 RT versus UC** | | | | | |
|  | HJ Jia 2018 | 1 | 116 | MD -4.23 (-5.86 to -2.60) | P<0.00001 |
|  | YH Gao 2019 | 1 | 80 | MD -9.05 (-11.02 to -7.08) | P<0.00001 |
|  | ***Subtotal*** | 2 | 196 | **MD -6.61 (-11.33 to -1.88)** | **P=0.006** |
| *Keep two decimal place. Abbreviation: CHD: coronary heart disease; RT: resistance training; AT: aerobic training; UC: usual care; Peak VO_2_: peak oxygen uptake; VO_2_ max: maximum oxygen uptake; QOL: quality of life; LVEF: left ventricular ejection fraction; LVEDD: left ventricular end-diastolic dimension. | | | | | |
