# Supplementary material for: Efficacy and Safety of Resistance Training for Coronary Heart Disease Rehabilitation: A Systematic Review of Randomized Controlled Trials
Source: Front Cardiovasc Med. 2021 Nov 5;8:754794. doi: 10.3389/fcvm.2021.754794 (PMC8602574; doi:10.3389/fcvm.2021.754794)
Supplement: Supplementary file 5 [file Data_Sheet_5.docx]

| **Appendix 5. Safety and adverse events reported** | | | | | |
| --- | --- | --- | --- | --- | --- |
| Study ID | No. of adverse events | | Effect estimate (95%CI) | P value | Adverse reactions |
|  | Experimental group | Control group |  |  |  |
| **1.Some adverse events** | | | | | |
| **1.1 RT+AT vs AT** | | | | | |
| B Dwiputra 2016 | 1/44 | 3/43 | RR 0.33 (0.04 to 3.01) | P=0.32 | T: Acute heart failure (1); C: Acute heart failure (1), Wound infection(2) |
| B Yael 1999 | 0/18 | 1/17 | RR 0.32 (0.01 to 7.26) | P=0.47 | C: arthritic knee pain (1) |
| CH Luan 2019 | 0/50 | 1/50 | RR 0.33 (0.01 to 7.99) | P=0.50 | C: percutaneous transluminal coronary intervention again (1) |
| FR Caruso 2017 | 1/13 | 1/12 | RR 0.92 (0.06 to 13.18) | P=0.95 | T: joint pain (1); C: joint pain (1) |
| MH Kelemen1986 | 9/20 | 0/20 | RR 19.00 (1.18 to 305.88) | P=0.04 | T: symptomatic hypotension and responded to the oral administration of fluids (1), brief episodes of ventricular bigeminy (4), isolated premature ventricular complexes (4) |
| PL M 2001 | 6/19 | 0/17 | RR 11.70 (0.71 to 193.38) | P=0.09 | T: low back pain (4), elbow tendonitis (1), shoulder pain (1) |
| ***Subtotal*** | *17/164* | *6/159* | *RR 1.39 (0.28 to 6.78)* | *P=0.69* | / |
| **1.2 RT vs AT** | | | | | |
| S Ghroubi 2013 | 3/16 | 7/16 | RR 0.43 (0.13 to 1.37) | P=0.15 | T: knee pain (3); C: knee pain (5), significant exercise-induced ST-segment depression without chest pain (2) |
| **1.3 RT vs UC** | | | | | |
| HJ Jia 2018 | 9/58 | 28/58 | RR 0.32 (0.17 to 0.62) | P=0.0007 | T: restenosis(4), angina(5); C: restenosis(10), angina(12), sinus arrhythmia(4), myocardial Infarction attack again(2) |
| KQ Wu 2017 | 22/30 | 29/34 | RR 0.86 (0.66 to 1.11) | P=0.25 | T: chest discomfort(22); C: chest discomfort(29) |
| SH Zhang 2018 | 12/30 | 32/34 | RR 0.39 (0.24 to 0.61) | P<0.0001 | T: angina (8), myocardial Infarction (1), ventricular arrhythmias (3); C: angina(17), myocardial Infarction (3), ventricular arrhythmias (9), heart failure (3) |
| ***Subtotal*** | *43/121* | *89/126* | *RR 0.49 (0.23 to 1.07)* | *P=0.07* | / |
| **2.Rehospitalization** | | | | | |
| **2.1 RT+AT vs AT** | | | | | |
| CH Luan 2019 | 1/50 | 2/50 | RR 0.50 (0.05 to 5.34) | P=0.57 | T: rehospitalization for heart failure (1);  C: rehospitalization for heart failure (2) |
| **2.2 RT vs UC** | | | | | |
| SH Zhang 2018 | 4/33 | 15/34 | RR 0.27 (0.10 to 0.74) | P=0.01 | T: rehospitalization (4);  C: rehospitalization (15) |
| YH Gao 2019 | 6/40 | 16/40 | RR 0.38 (0.16 to 0.86) | P=0.02 | T: rehospitalization (6);  C: rehospitalization (16) |
| ***Subtotal*** | *10/73* | *31/74* | *RR 0.33 (0.17 to 0.62)* | *P=0.0006* | / |
| Abbreviation: T: Experimental group; C: Control group. | | | | | |
